# Diploid hepatocytes resist acetaminophen-induced liver injury through suppressed JNK signaling

**DOI:** 10.1038/s41419-026-08448-z

**Published:** 2026-02-03

**Authors:** Sierra R. Wilson, Evan R. Delgado, Frances Alencastro, Rosa L. Loewenstein, Madeleine P. Leek, Leah R. Peters, Kerollos Kamel, Patrick D. Wilkinson, Siddhi Jain, Joseph Locker, Silvia Liu, Bharat Bhushan, Andrew W. Duncan

**Affiliations:** 1 Department of Pathology, University of Pittsburgh School of Medicine, University of Pittsburgh, Pittsburgh, PA USA; 2 McGowan Institute for Regenerative Medicine, University of Pittsburgh School of Medicine, University of Pittsburgh, Pittsburgh, PA USA; 3 Pittsburgh Liver Research Center, University of Pittsburgh School of Medicine, University of Pittsburgh, Pittsburgh, PA USA; 4 Regenerative Medicine and Cell Biology, College of Medicine, Medical University of South Carolina, Charleston, SC USA; 5 Organ Pathobiology and Therapeutics Institute, University of Pittsburgh School of Medicine, University of Pittsburgh, Pittsburgh, PA USA; 6 Department of Pharmacology and Chemical Biology, University of Pittsburgh School of Medicine, University of Pittsburgh, Pittsburgh, PA USA; 7 Department of Bioengineering, School of Engineering, University of Pittsburgh, Pittsburgh, PA USA

**Keywords:** Pathogenesis, Chromosomes, Disease model, Cell proliferation, Cell death

## Abstract

The liver contains both diploid and polyploid hepatocytes, but their functional differences remain poorly understood. Emerging evidence suggests that each ploidy state contributes to regeneration in an injury-specific manner. We hypothesized that diploid hepatocytes promote healing after acetaminophen (APAP)-induced liver injury. To study ploidy populations in vivo, we utilized mice with a lifelong liver-specific knockout of *E2f7*/*E2f8* (LKO), which are enriched in diploid hepatocytes (> 70%) but otherwise normal. Control and LKO mice were treated with APAP (300 or 600 mg/kg), and injury was assessed over 0–96 h. Although both groups sustained injury, LKO mice showed improved survival, lower serum liver enzyme levels, and reduced necrosis and DNA fragmentation, indicating resistance to APAP-induced injury. To determine if resistance was due to *E2f7/E2f8* loss or increased diploidy, we deleted *E2f7/E2f8* in adult hepatocytes (HKO), a model that does not alter ploidy. Injury was similar between controls and HKO, ruling out gene deletion as the protective factor. Transcriptomic and protein analyses revealed minimal baseline differences; however, following APAP treatment, LKO livers exhibited reduced JNK activation and less mitochondrial injury. Finally, APAP-treated wild-type hepatocytes exhibited a shift toward lower ploidy, supporting the idea that diploid cells are more resistant to injury. These findings highlight hepatocyte ploidy as a key determinant of injury response and suggest a protective role for diploid hepatocytes in promoting liver resilience and regeneration.

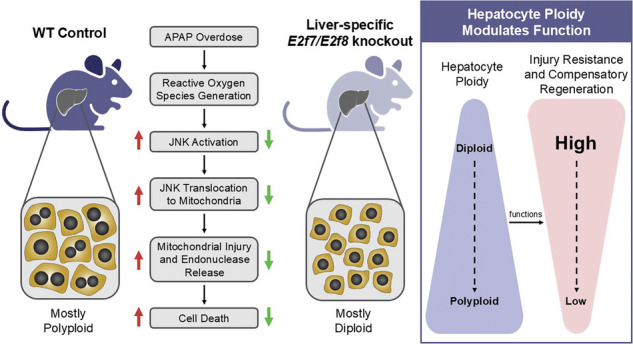

## Introduction

Most mammalian somatic cells are diploid, containing two copies of each chromosome set. However, certain cell types, including hepatocytes, undergo polyploidization, resulting in cells with multiple sets of chromosomes. The liver is a uniquely polyploid organ with polyploid hepatocytes comprising ~90% of the liver in adult mice and 20–50% in humans [1, 2]. Polyploidization primarily occurs during postnatal development through acytokinetic mitosis [3–8]. Hepatic polyploidy can also be reversed, generating lower-ploidy daughters that can then re-polyploidize [1, 9]. The regulation of hepatocyte polyploidization, ploidy reversal, and re-polyploidization, is controlled by a network of signaling pathways that influence cell cycle progression and cytokinesis [3, 4, 10].

Although the mechanisms underlying hepatocyte polyploidization are well defined, the functions of distinct ploidy subpopulations remain unclear. Emerging data suggest that diploid and polyploid hepatocytes play distinct roles in liver physiology and disease. For example, excessive polyploidy is observed in metabolic dysfunction-associated steatotic liver disease and steatohepatitis, where hepatocytes show abnormal increases in mononucleated, high-ploidy cells [11, 12]. The consequences of hyperpolyploidy are unclear, with potential roles in both promoting and protecting against disease. Polyploid hepatocytes can also undergo ploidy reversal, generating diploid or aneuploid progeny that enhance genetic diversity and promote adaptation to chronic liver injury. In mouse models of tyrosinemia, such aneuploid descendants clonally expand to repopulate the liver, conferring a selective advantage during injury [1, 13–15]. This highlights a role for polyploid-derived aneuploidy in providing adaptive genetic diversity. In hepatocellular carcinoma (HCC), ploidy plays a nuanced role. Most human and rodent HCCs are diploid, suggesting increased susceptibility of diploid hepatocytes to malignant transformation. Supporting this, polyploid hepatocytes can suppress tumor initiation, particularly when tumor suppressor genes are lost, by harboring extra copies of chromosomes that provide redundant tumor suppressor function [16]. Once transformed, diploid hepatocytes tend to proliferate extensively, driving tumor growth [17]. However, polyploid hepatocytes can undergo ploidy reduction, giving rise to diploid progeny that contribute to tumorigenesis [9]. Moreover, polyploid HCCs have been identified in humans, particularly in tumors harboring *TP53* mutations [2].

Our group recently demonstrated that hepatocyte ploidy influences proliferative capacity, with diploid hepatocytes exhibiting a distinct proliferative advantage over polyploids. Liver-specific knockout of *E2f7/E2f8* (LKO) mice develop mature livers with hepatocytes that are predominantly diploid, and they maintain normal liver function into adulthood, making them a valuable tool to investigate ploidy [17–20]. In competitive repopulation assays, hepatocytes from highly diploid *E2f7/E2f8* LKO mice were co-transplanted with mostly polyploid wild-type (WT) hepatocytes into *Fah*⁻^/^⁻ recipients. Diploid-enriched hepatocytes consistently outcompeted polyploids during liver repopulation, indicating enhanced ability to proliferate under selective pressure. Similarly, in response to regenerative signals in WT mice (e.g., after partial hepatectomy or growth factor stimulation in vitro), diploid hepatocytes entered and progressed through the cell cycle more rapidly than polyploids. This enhanced proliferation by diploids was also observed in humans using retrospective radiocarbon birth dating, which revealed that diploid hepatocytes have a higher turnover rate than polyploids, suggesting that human hepatocyte proliferation is driven by diploids [21].

We hypothesized that diploid-enriched livers would exhibit enhanced regeneration and protection after acute liver injury. To test this hypothesis, we used a well-characterized model of acetaminophen (APAP)-induced liver injury. APAP overdose is the leading cause of acute liver failure in the United States, accounting for nearly 50% of cases annually [22]. Although APAP is safe at therapeutic doses, excessive intake leads to accumulation of its toxic metabolite, N-acetyl-p-benzoquinone imine (NAPQI) [23]. NAPQI is typically detoxified by conjugation with glutathione (GSH). However, during overdose, GSH stores are rapidly depleted, allowing NAPQI to form adducts with mitochondrial proteins, initiating oxidative stress and mitochondrial dysfunction [24–26]. This triggers a cascade of cellular events, including c-Jun N-terminal kinase (JNK) activation, amplifying mitochondrial damage and necrotic cell death [27, 28]. While mild overdoses may be resolved through spontaneous liver regeneration, severe injury can result in irreversible damage and progression to liver failure, where liver transplantation is the only treatment option.

Here, we investigated how hepatocyte ploidy affects susceptibility to APAP-induced liver injury by comparing diploid-enriched LKO and polyploid-enriched control mice. We found that LKO mice resisted APAP-induced liver injury and initiated hepatocyte proliferation earlier than controls. This protective phenotype was associated with reduced JNK activation and diminished mitochondrial damage. Further, in WT hepatocytes, diploids were more resistant to APAP toxicity than polyploids. Together, these findings identify diploid hepatocytes as a protective subpopulation that promotes survival through stress signaling and regenerative dynamics.

## Results

### Liver-specific *E2f7/E2f8* deletion confers resistance to APAP-induced hepatotoxicity

**Fig. 1:**
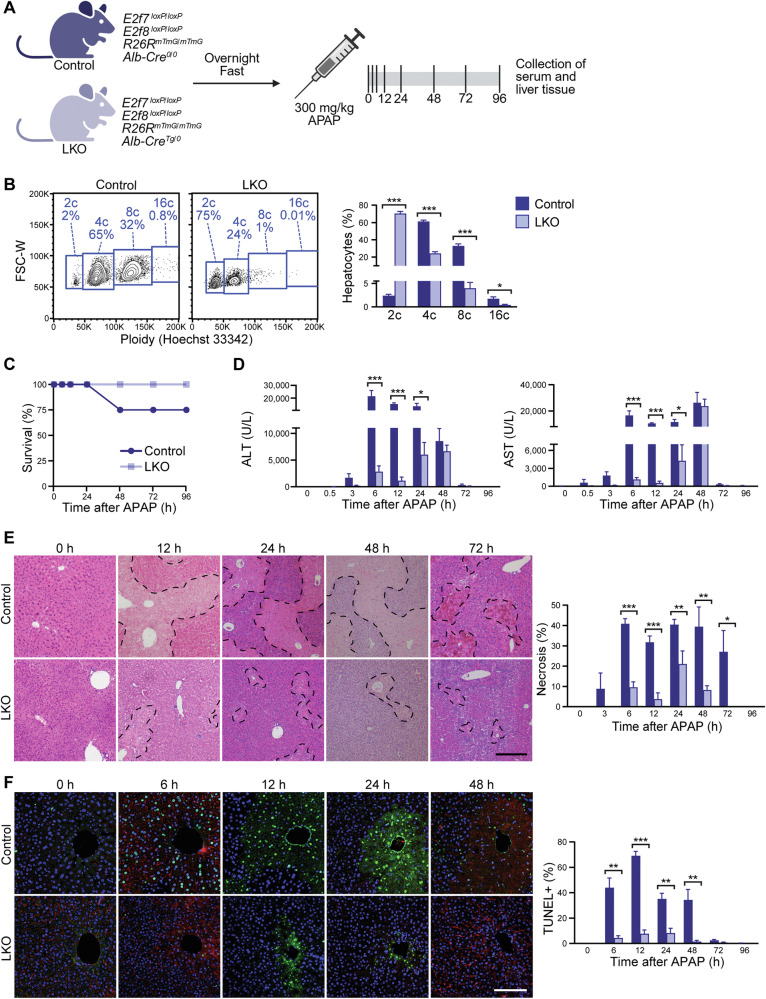
Liver-specific *E2f7/E2f8* deletion produces “polyploid knockout” mice resistant to APAP-induced hepatotoxicity. **A** Experimental scheme (Created with BioRender.com). **B** Freshly isolated hepatocytes from 3-month-old control and LKO mice were stained with Hoechst 33342 to determine cellular ploidy of viable hepatocytes by flow cytometry. Representative flow plots show the distribution of diploid (2c), tetraploid (4c), octaploid (8c), and hexadecaploid (16c) populations (*n* = 5–7/genotype; males). The full FACS gating strategy is shown in Supplementary Fig. S1B. **C** Survival curves of control and LKO mice following APAP overdose (n = 4–10/genotype/timepoint). **D** Levels of liver biomarkers ALT and AST in the serum (*n* = 4–9/genotype/timepoint). **E** Quantification of necrosis by H&E staining (*n* = 4–9/genotype/timepoint). Scale bar = 200 µM. **F** TUNEL staining showing DNA fragmentation (green) and nuclear staining with Hoechst 33342 (blue) (*n* = 4–9/genotype/timepoint). Scale bar = 100 µM. Representative images, plots, and quantification results are shown. Graphs show mean ± SEM. *P < 0.05; **P < 0.01; ***P < 0.001.

To investigate how hepatocyte ploidy influences acute liver injury, we used lifelong, Alb-Cre-driven liver-specific *E2f7/E2f8* knockout (LKO) mice. In contrast to control livers that are predominantly polyploid, with only 2–5% diploid hepatocytes, LKO livers have >70% diploid hepatocytes and exhibit a corresponding reduction in hepatocyte cell area (Fig. 1A, B and Supplementary Fig. S1A, B). Mice were fasted, administered APAP, and harvested after 0–96 h (Fig. 1A). We first examined the response to 300 mg/kg APAP, characterized as a “regenerating dose” that induces injury followed by regeneration and recovery [29]. Twenty-five percent of control mice died, while LKO mice survived completely, suggesting resistance to APAP-induced damage (Fig. 1C). Similarly, serum levels of the liver injury biomarkers ALT and AST were lower in LKO mice compared to controls, between 6 and 24 h after injury (Fig. 1D). Histological examination revealed widespread centrilobular necrosis in control livers, while LKO livers displayed reduced necrosis from 6 to 72 h (Fig. 1E). TUNEL staining showed reduced DNA fragmentation in LKO livers, further demonstrating APAP resistance by the LKO model (Fig. 1F).

To test whether LKO mice retained this protective phenotype under more stringent conditions, we challenged them with 600 mg/kg APAP, a “non-regenerating dose” that causes sustained hepatocellular injury, impaired regeneration, and poor survival outcomes [29]. The mortality of control mice increased dramatically, with 91% dying within 72 h (Supplementary Fig. S2A). Strikingly, 53% of LKO mice survived, highlighting a survival advantage. Among the surviving animals, ALT and AST levels were comparable between genotypes, while necrosis and DNA fragmentation were reduced in LKO mice at 48 and 24 h, respectively (Supplementary Fig. S2B–D). Because only 9% of control mice survived to 72 h, the samples collected at this time point represent rare survivors with less severe injury. As a result, ALT/AST values likely underestimate the true extent of hepatotoxicity and reflect survivor bias rather than reduced injury. Together, these results demonstrate that diploid-enriched LKO mice significantly resist APAP-induced liver injury.

### Diploid-rich LKO livers exhibit enhanced proliferation

**Fig. 2:**
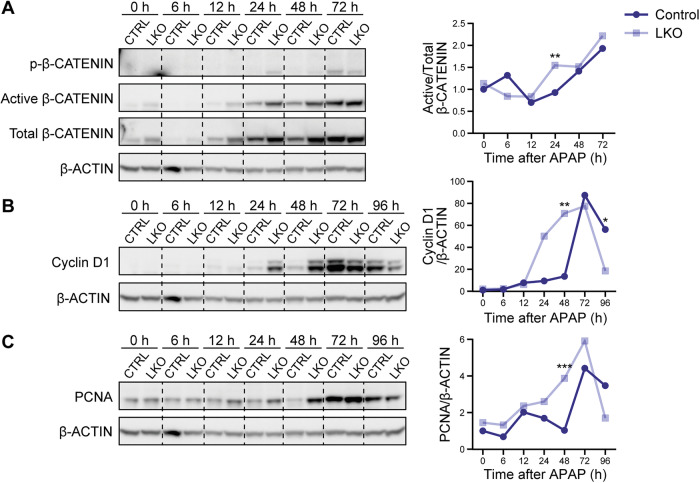
LKO hepatocytes display enhanced proliferation after APAP overdose. Western blotting of whole liver lysates collected from control and LKO mice 0–96 h after 300 mg/kg APAP overdose, showing expression of proteins involved in proliferation: **A** active β-CATENIN relative to total β-CATENIN (pooled samples; *n* = 3/genotype/timepoint), **B** Cyclin D1 relative to β-ACTIN (pooled samples; *n* = 3/genotype/timepoint), **C** PCNA relative to β-ACTIN (pooled samples; *n* = 3/genotype/timepoint). Shown are representative blots and quantification results normalized to the 0 h control, which is set to 1. The significance values are based on western blot analysis of individual samples shown in Supplementary Fig. S2 (*n* = 3–5/genotype/timepoint). *P < 0.05; **P < 0.01; ***P < 0.001.

We next examined whether LKO mice exhibit altered regenerative responses following 300 mg/kg APAP injury. Whole liver lysates were analyzed for proteins involved in proliferation. Levels of active β-CATENIN (non-phosphorylated at Ser45), which directly promotes expression of the G1/S regulator Cyclin D1, were comparable between groups at baseline and up to 12 h post-injury. By 24 h, LKO mice showed elevated active β-CATENIN compared to controls, suggesting that diploid hepatocytes in LKO livers initiate cell cycle entry earlier (Fig. 2A, pooled samples; Supplementary Fig. S3A, individual replicates). This trend is mirrored by Cyclin D1 and PCNA, which increase by 48 h after APAP overdose, indicating compensatory regeneration occurs, with significantly increased expression in the LKO at 48 h. By 96 h, the pattern is reversed, with control mice displaying higher Cyclin D1 and PCNA (Fig. 2B, C and Supplementary Fig. S3B). To assess whether differences in β-CATENIN activation were due to altered degradation, we examined phosphorylated β-CATENIN (Ser33/37/Thr41), which targets the protein for proteasomal degradation, and found no differences between groups (Fig. 2A, pooled samples; Supplementary Fig. S3A, individual replicates). Thus, despite sustaining less liver injury after APAP injection, LKO mice expressed proliferation markers at levels comparable to or greater than those of controls. This finding suggests that diploid hepatocytes in LKO livers not only resist APAP-induced injury but also initiate regeneration more rapidly.

### *E2f7/E2f8* deletion alone does not confer resistance to APAP-induced injury

**Fig. 3:**
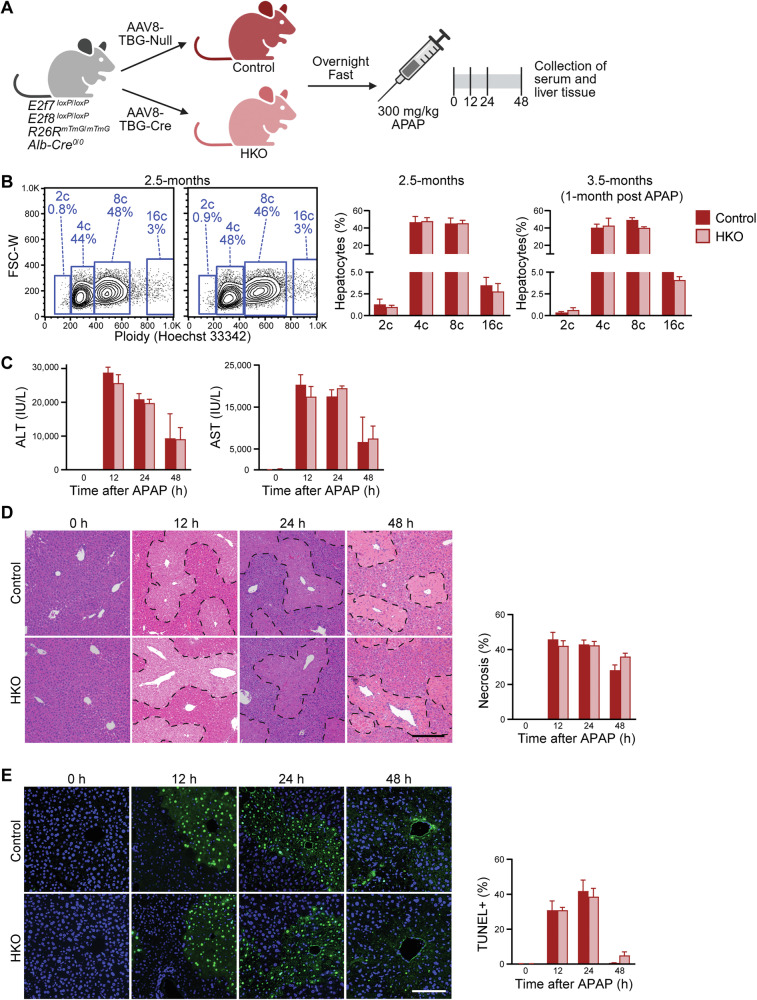
Loss of *E2f7/E2f8* in adults with normal ploidy does not protect against APAP-induced damage. **A** Experimental scheme (Created with BioRender.com). **B** Ploidy analysis by flow cytometry of freshly isolated hepatocytes from 2.5-month-old control and HKO mice at baseline, and from 3.5-month-old control and HKO mice 1 month after 300 mg/kg APAP treatment and recovery. (*n* = 3/genotype/timepoint; males). **C** Levels of liver biomarkers ALT and AST in the serum (*n* = 3–6/genotype/timepoint). **D** Quantification of necrosis by H&E staining (*n* = 3–6/genotype/timepoint). Scale bar = 200 µM. **E** TUNEL staining showing DNA fragmentation (green) and nuclear staining with Hoechst 33342 (blue) (*n* = 3–6/genotype/time point). Scale bar = 100 µM. Representative images, plots, and quantification results are shown. Graphs show mean ± SEM. *P < 0.05; **P < 0.01; ***P < 0.001.

To determine whether APAP resistance in LKO mice was due to gene expression changes associated with *E2f7*/*E2f8* deletion or the enrichment of diploid hepatocytes in the LKO model, we generated a hepatocyte-specific knockout (HKO) model by injecting adult mice (containing loxP sites in *E2f7* and *E2f8*) with AAV8-TBG-Cre to delete *E2f7/E2f8*. Control animals received AAV8-TBG-Null virus. Because adult livers are already predominantly polyploid, this approach enables deletion of *E2f7/E2f8* without altering ploidy. Two weeks after AAV8 injection, mice were fasted, administered 300 mg/kg APAP, and harvested at multiple time points (Fig. 3A).

To evaluate the HKO model, we examined the R26R-mTmG reporter. Two weeks after AAV8 injection, all hepatocytes in control livers were tdTomato^+^, while >99% of hepatocytes in HKO livers were GFP^+^, indicating efficient Cre-mediated recombination with no change in overall hepatocyte size (Supplementary Fig. S4A). Next, to assess transcriptomic differences between control and HKO mice, we performed RNA sequencing on whole livers at 0 h. *E2f8* expression was reduced in HKO mice, indicating efficient gene deletion (Supplementary Fig. S4B). *E2f7* is lowly expressed in adult livers and was below detection. Transcriptomic analysis revealed 320 differentially expressed genes between HKO and control, suggesting modest changes (Supplementary Fig. S4C and Supplementary Table S1). Finally, ploidy analysis demonstrated that control and HKO livers remained predominantly polyploid at baseline (Fig. 3B). Moreover, one month following APAP treatment, when livers had recovered from APAP overdose, the HKO ploidy spectrum remained equivalent to controls, indicating that loss of *E2f7*/*E2f8* in adults does not affect ploidy even after extensive compensatory liver regeneration (Fig. 3B).

Serum ALT and AST levels, necrosis, and DNA fragmentation were comparable between control and HKO mice after overdose (Fig. 3C–E). Key proliferative signaling markers, active β-CATENIN, Cyclin D1, and PCNA, were expressed similarly between HKO and controls at most time points, with only minor differences observed (Supplementary Fig. S4D, E). Thus, in contrast to the LKO model, HKO mice showed no alterations in ploidy or injury after APAP. These findings indicate that the resistance to APAP-induced toxicity observed in LKO mice is independent of *E2f7/E2f8* deletion and is likely attributed to the high percentage of diploid hepatocytes.

### APAP metabolism machinery is conserved in the LKO model

**Fig. 4:**
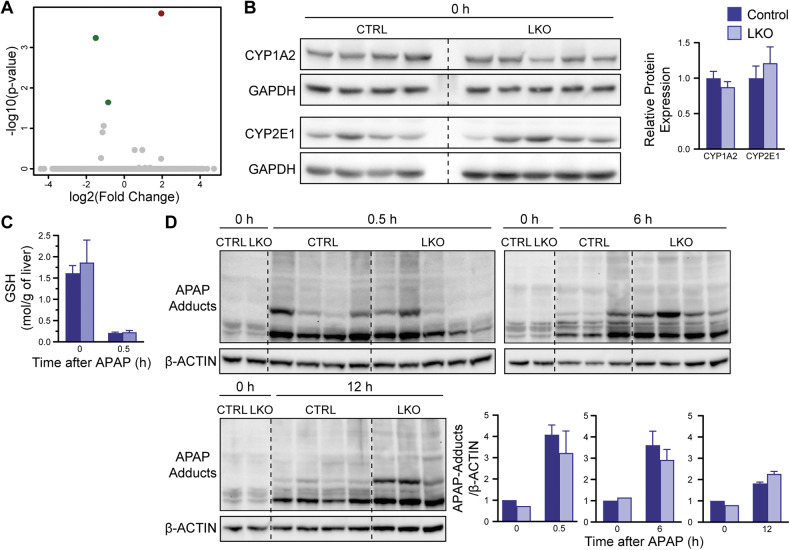
APAP metabolism machinery is conserved in the LKO model. **A** Volcano plot showing differential gene expression between control and LKO mice at baseline (0 h; *n* = 4/genotype). Differentially expressed genes were defined by FDR = 5% and fold change ≥ 1.5. See Supplementary Data Table S2 for gene list. **B** Expression levels of key APAP-metabolizing enzymes CYP2E1 and CYP1A2 in control and LKO mice at baseline (*n* = 4–5/genotype/timepoint). **C** Baseline hepatic GSH levels and its depletion 0.5 h after 300 mg/kg APAP overdose in control and LKO mice (*n* = 4–6/genotype/timepoint). **D** Formation of APAP-protein adducts at 0, 0.5, 6, and 12 h following 300 mg/kg APAP (*n* = 3–5/genotype/timepoint). Graphs show mean ± SEM. *P < 0.05; **P < 0.01; ***P < 0.001.

To identify the mechanism driving APAP resistance by diploid hepatocytes, we turned to the LKO model. Bulk RNA-seq of livers from control and LKO mice that were fasted but untreated (0 h time point) revealed few differentially expressed genes, consistent with previous reports (Fig. 4A and Supplementary Table S2) [20]. Given the minimal transcriptional differences observed, we next examined protein levels of enzymes involved in APAP metabolism to determine whether protein expression could account for the observed phenotype. Cytochrome P450 enzymes, CYP2E1 and CYP1A2, key mediators of APAP bioactivation to the hepatotoxic metabolite NAPQI, were unchanged between LKO and control livers at baseline (Fig. 4B). We next examined hepatic GSH levels, the major detoxifying molecule that neutralizes the toxic APAP metabolite NAPQI. GSH concentrations at 0 h and 30 min after 300 mg/kg APAP were similar, indicating that LKO and control mice have comparable capacity for detoxification (Fig. 4C). Additionally, APAP-protein adduct formation was similar between LKO and controls after 0.5, 6, and 12 h (Fig. 4D). Thus, APAP metabolism and detoxification are intact in both groups.

**Fig. 5:**
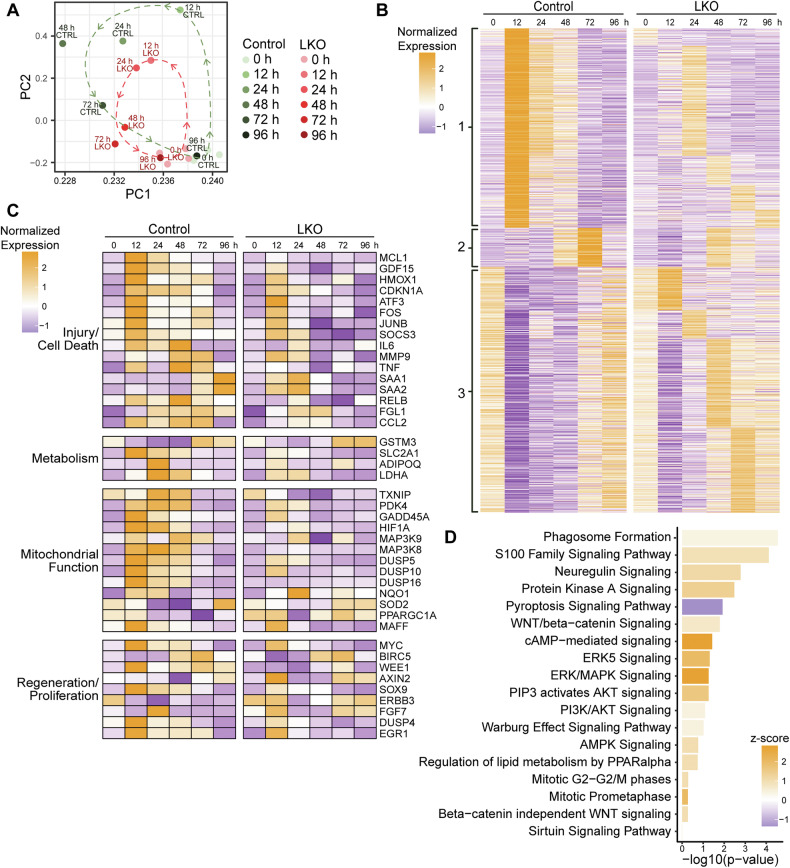
Gene expression in control and LKO livers. **A** PCA of RNA-seq data. Control samples (green) and LKO samples (red) are shown (0 h *n* = 4/genotype; 12–96 h samples are pooled with *n* = 3/genotype/timepoint). **B** Heat map showing expression patterns of differentially expressed genes grouped into clusters 1-3 based on temporal expression patterns. For example, genes in cluster 1, which were rapidly upregulated in control livers within 12 h of APAP administration, showed delayed or absent induction in LKO livers. In contrast, genes in cluster 3 that were induced later (72-96 h) in controls appeared to be activated earlier in LKO livers, suggesting a dysregulated injury response. See Supplementary Table S3 for gene lists. **C** Heat map of differentially expressed genes categorized by functional pathways, including injury/cell death, metabolism, mitochondrial function, and regeneration/proliferation. See Supplementary Table S4 for gene lists. **D** IPA of control and LKO livers at 12 h post-APAP (pooled samples; *n* = 3/genotype). Relevant enriched pathways are shown.

Although few gene expression differences were observed at baseline, RNA-seq analysis at 12–96 h after APAP overdose revealed divergent transcriptional responses between control and LKO mice. Principal component analysis (PCA) showed that samples from both groups clustered tightly together at 0 h (Fig. 5A). However, following APAP injury, the two groups separated along principal components, indicating distinct transcriptional trajectories. Control livers followed a broad arc, with gene expression divergence peaking between 12 and 24 h. In contrast, LKO livers show a limited change in gene activity that remained distinct from controls. By 96 h, the two groups moved closer together toward the 0 h time point, suggesting resolution of injury.

Broad, temporal changes in gene expression were observed between control and LKO mice after APAP overdose (Fig. 5B and Supplementary Table S3). Strikingly, key differentially expressed genes included those involved in cellular injury, oxidative stress, mitochondrial function, and proliferation (Fig. 5C and Supplementary Table S4). One key example is *Cdkn1a*, which encodes p21, a regulator of cell cycle arrest and DNA damage response. In controls, *Cdkn1a* was strongly upregulated and remained elevated for a longer duration than in LKO, consistent with a more sustained injury response. Ingenuity Pathway Analysis (IPA) identified the activation of WNT/β-catenin signaling in LKO livers (Fig. 5D), aligning with increased protein levels of active β-CATENIN and its downstream target Cyclin D1 (Fig. 2 and Supplementary Fig. S3). Sirtuin signaling, a pathway involved in oxidative stress responses and previously linked to APAP toxicity, was also predicted to be activated in LKO mice [30–34]. However, protein levels of SIRT1, SIRT3, and SIRT6 at 0 and 6 h post-APAP were similar between groups (Supplementary Fig. S5), suggesting that Sirtuins do not account for the observed hepatoprotection.

Together, these results demonstrate that LKO mice have intact APAP metabolism and detoxification capacity, indicating that their resistance to APAP-induced liver injury is not due to differences in early drug metabolism. Instead, the findings point to altered injury and recovery responses in LKO livers. This suggests that diploid and polyploid hepatocytes respond differently to stress, with diploids potentially mounting a more protective or regenerative transcriptional response following APAP injury.

### Blunted JNK activation mediates hepatoprotection in LKO mice

**Fig. 6:**
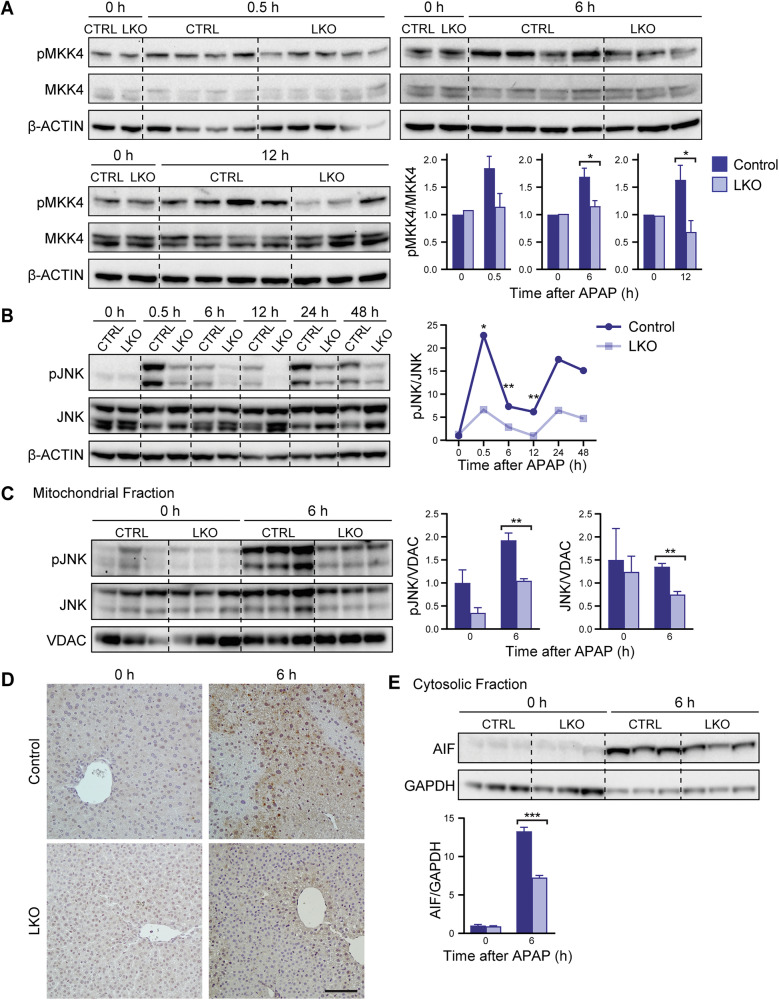
APAP resistance in the LKO model is mediated by JNK activation. **A** Expression of pMKK4 relative to total MKK4 in whole liver lysates from control and LKO mice 0–12 h after 300 mg/kg APAP (*n* = 3–5/genotype/timepoint). **B** Expression of pJNK relative to total JNK in whole liver lysates from control and LKO mice 0–48 h after APAP. Samples are pooled (*n* = 3/genotype/timepoint). The significance values are based on Western blot analysis of individual samples shown in Supplementary Fig. S6A. **C** Expression of pJNK and total JNK relative to VDAC in the mitochondrial fraction of control and LKO livers 0 and 6 h after APAP (*n* = 3/genotype/timepoint). **D** Representative nitrotyrosine staining (brown) in control and LKO liver sections collected 0 and 6 h following APAP exposure. Scale bar = 50 µM. **E** Expression of cytosolic AIF in control and LKO livers 0 and 6 h after APAP (*n* = 3–5/genotype/timepoint). Shown are representative blots and quantification results normalized to the 0 h control, which is set to 1. Graphs show mean ± SEM. *P < 0.05; **P < 0.01; ***P < 0.001.

Considering the pattern of attenuated expression of genes related to cell death and injury combined with preserved mitochondrial function in the LKO mice, we next examined the JNK signaling pathway, a central mediator of APAP-induced mitochondrial dysfunction and necrotic cell death. Activation of JNK is initiated by upstream kinases like MKK4, which phosphorylate JNK and facilitate its translocation to the mitochondria, where it exacerbates mitochondrial oxidative stress and promotes necrotic cell death [27, 35, 36]. Western blot analysis revealed that active MKK4 (pMKK4) was significantly reduced in LKO livers at 6 and 12 h after APAP overdose (Fig. 6A). Consistent with reduced MKK4 activity, phosphorylated JNK (pJNK) levels were also decreased in whole-liver lysates from LKO mice across multiple timepoints (Fig. 6B, pooled samples; Supplementary Fig. S6A, individual replicates). Similarly, mitochondrial fractions isolated 6 h post-injury contained markedly less total and phosphorylated JNK in LKO mice, indicating reduced JNK translocation to mitochondria (Fig. 6C).

To evaluate early mitochondrial oxidative stress, we first assessed nitroytrosine formation, a well-established marker of peroxynitrite-mediated protein nitration that precedes permeability transition and cell death [37, 38]. Nitrotyrosine staining was reduced in LKO livers 6 h after APAP treatment (Fig. 6D), indicating lower levels of mitochondrial oxidative burden at this early stage of injury. We next examined a downstream effect of sustained mitochondrial damage by measuring cytosolic levels of apoptosis-inducing factor (AIF), a mitochondrial endonuclease that is released after the mitochondrial permeability transition and contributes to DNA fragmentation during necrotic cell death. At 6 h post-APAP, LKO livers showed lower cytosolic AIF levels than controls (Fig. 6E), consistent with reduced activation of the mitochondrial permeability transition and preserved mitochondrial integrity.

This blunted stress response in LKO mice was further supported by transcriptomic profiling in Fig. 5. Oxidative stress-responsive genes such as *Txnip* and *Nqo1* were strongly induced in control livers after APAP overdose but showed blunted or delayed activation in LKO mice (Fig. 5C), consistent with reduced oxidative pressure. Upstream regulators of JNK signaling, including *Map3k8* and *Map3k9*, were also downregulated in LKO livers (Fig. 5C), consistent with decreased MKK4 and JNK phosphorylation. Together, RNA and protein expression patterns support the idea that diploid-enriched LKO livers exhibit reduced JNK activation and, consequently, less mitochondrial injury following APAP overdose.

To determine whether reduced JNK activation arises from increased diploidy or from deletion of *E2f7/E2f8*, we evaluated pJNK levels in HKO mice following APAP treatment. Unlike LKO mice, HKO showed no reduction in JNK activation compared to controls (Supplementary Fig. S6B), suggesting that reduced JNK activation in LKO is not a direct consequence of *E2f7*/*E2f8* loss but is instead likely driven by the enrichment of diploid hepatocytes. Taken together, these data support a model in which reduced JNK activation and diminished mitochondrial stress signaling drive the resistance to APAP toxicity observed in diploid hepatocytes.

### Highly polyploid hepatocytes are sensitive to APAP toxicity

**Fig. 7:**
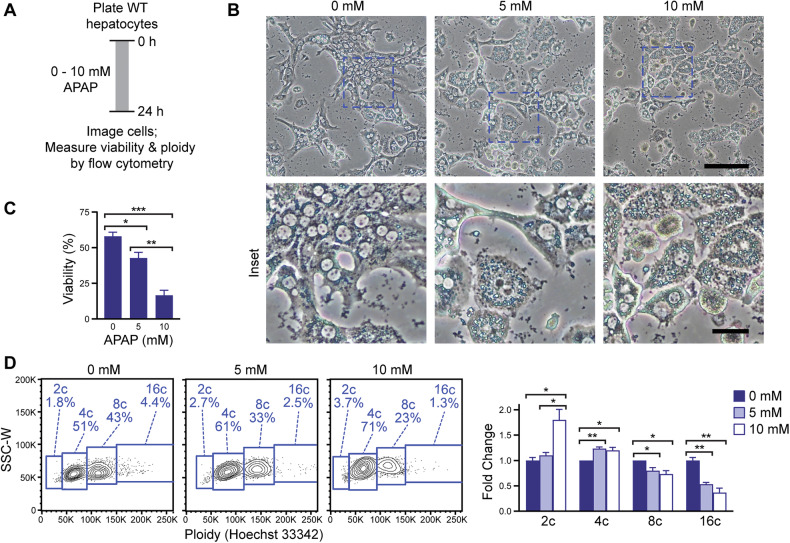
Highly polyploid hepatocytes are more susceptible to APAP-induced cell death. **A** Schematic of in vitro APAP toxicity assay. Primary hepatocytes were isolated from 2–3-month-old WT mice (male), treated with increasing doses of APAP (0, 5, and 10 mM) for 24 h. **B** Representative images showing APAP-induced dose-dependent hepatocyte injury at 24 h; higher-magnification insets highlight cellular detail (main image scale bar = 100 µm; inset scale bar = 25 µm). **C** Quantification of cell death by FVD staining. **D** Flow cytometry plots showing relative ploidy distribution (2c, 4c, 8c, 16c) within viable (FVD negative) hepatocytes. Data are shown as the fold change of each ploidy population, where 0 mM APAP was set to 1. Data shown have *n* = 3 internal replicates per condition and are representative of 5 independent experiments. Graphs show mean ± SEM. *P < 0.05; **P < 0.01; ***P < 0.001.

To determine whether the APAP resistance observed in diploid-enriched LKO mice is a general feature of diploid hepatocytes, we evaluated APAP sensitivity in WT hepatocytes. Primary hepatocytes were isolated from adult C57BL/6J mice, treated with increasing concentrations of APAP, and assessed after 24 h (Fig. 7A). Microscopy revealed a clear dose-dependent increase in cellular injury, including hepatocyte rounding, lipid droplet accumulation, and cell death (Fig. 7B). To quantitatively assess cell death, hepatocytes were stained with a fixable viability dye (FVD) and Hoechst 33342, allowing for the simultaneous measurement of viability and ploidy using flow cytometry. As expected, APAP treatment led to a dose-dependent increase in cell death, with hepatic viability decreasing from 58% at 0 mM APAP to 17% at 10 mM (Fig. 7C).

To investigate whether ploidy influences susceptibility to APAP-induced death, we analyzed the distribution of hepatocyte ploidy in viable populations. In untreated cultures (0 mM), the expected distribution of 2c, 4c, 8c, and 16c hepatocytes was observed. However, following treatment with 10 mM APAP, when substantial cell death was present, there was a consistent decrease in the proportion of live 8c and 16c hepatocytes and an enrichment of 2c and 4c populations. For instance, treatment with 10 mM APAP led to a reduction in viable high-ploidy hepatocytes, with 8c cells decreasing from 40% to 29%, and 16c cells from 3.5% to 1.5%. In contrast, low-ploidy populations increased, with 2c hepatocytes doubling from 2% to 4% and 4c cells rising from 51% to 63% (Fig. 7D). Together, these data demonstrate that hepatocyte sensitivity to APAP-induced toxicity is influenced by ploidy status, with low-ploidy cells less susceptible to injury and death. These findings support the in vivo observation that diploid-enriched livers are more resistant to APAP and suggest that reduced ploidy states may confer a protective advantage in the context of acute hepatotoxic stress.

## Discussion

The liver is characterized by extensive hepatocyte polyploidy, yet the functional significance of different ploidy states remains incompletely understood. Here, we demonstrate that hepatocyte ploidy modulates the liver’s response to acute drug-induced injury. Diploid-enriched LKO mice exhibited reduced serum liver enzyme levels, diminished histological damage, and lower DNA fragmentation following APAP overdose. Despite sustaining less injury, LKO livers mounted an earlier and more robust regenerative response. These findings align with our earlier work [17] that diploid hepatocytes are primed for proliferation and extend this concept by revealing a previously unrecognized role in injury resistance. To validate the link between ploidy and APAP resistance, WT hepatocytes were challenged with APAP. Highly polyploid cells (8c, 16c) were significantly more sensitive to APAP-induced death than low-ploidy cells (2c, 4c), confirming that ploidy directly influences toxicity.

**Fig. 8:**
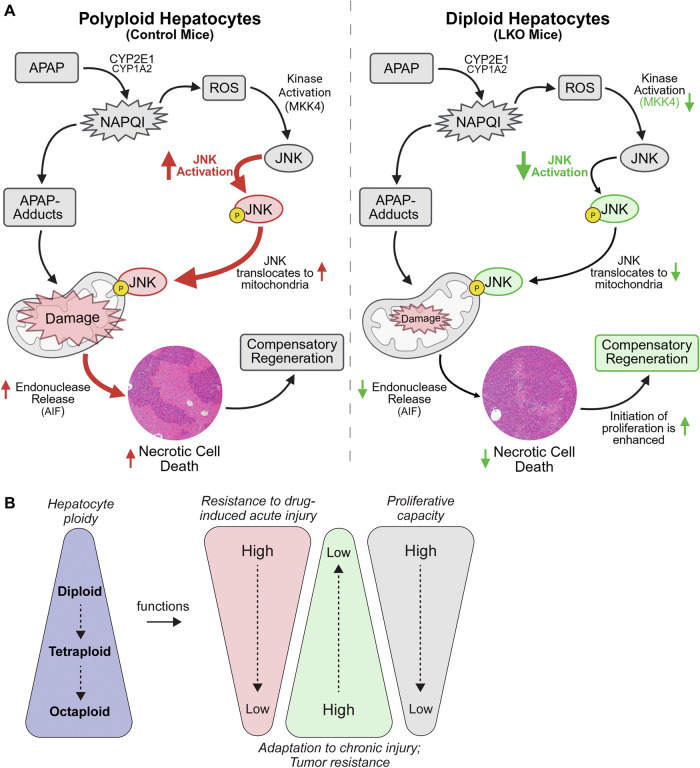
Summary of the ploidy-dependent response to APAP overdose in the liver. **A** Schematic summary comparing how polyploid (control) and diploid-enriched (LKO) livers respond to APAP overdose (Created with Biorender.com). Both cell types metabolize APAP through cytochrome P450 enzymes (CYP2E1, CYP1A2), generating the toxic intermediate NAPQI and forming APAP-protein adducts. In polyploid hepatocytes, this leads to oxidative stress, JNK activation and mitochondrial translocation, release of AIF, and necrotic cell death. In contrast, diploid hepatocytes suppress MKK4/JNK signaling activation and mitochondrial injury, limiting cell death. **B** Model illustrating context-dependent advantages of hepatocyte ploidy states. Diploid hepatocytes exhibit enhanced resistance to acute toxic injury, such as APAP overdose, and demonstrate a greater capacity for proliferation. In contrast, polyploid hepatocytes support tumor suppression and adaptation to chronic injury through mechanisms such as aneuploidy tolerance and metabolic buffering. This functional specialization enables the liver to respond flexibly across diverse injury contexts.

Resistance in LKO livers was not due to altered APAP metabolism. Expression of key cytochrome P450 enzymes (CYP2E1/CYP1A2), GSH depletion, and APAP-protein adduct formation were similar between LKO and controls. Instead, transcriptomic profiling revealed a distinct injury response in LKO livers, marked by reduced activation of injury-related genes and earlier induction of regeneration, including WNT/β-catenin signaling and Cyclin D1 expression. Strikingly, there were profound differences in JNK signaling between LKO and control livers (Fig. 8A). JNK is a well-established driver of APAP-induced hepatocyte necrosis, and its suppression was a key distinguishing feature of the diploid-enriched response. In LKO mice, reduced MKK4 phosphorylation led to lower JNK activation and mitochondrial translocation, along with decreased nitrotyrosine formation (early oxidative stress) and reduced cytosolic AIF release (later mitochondrial injury). These findings, which align with previous reports that pharmacologic inhibition and germline knockout of JNK protect against APAP toxicity [27, 28, 36, 39–42], support a model where diploid hepatocytes resist injury through suppression of JNK-mediated cell death.

Spatial zonation within the liver lobule is a critical consideration in APAP-induced injury, which targets pericentral hepatocytes with high CYP2E1 expression. If polyploid hepatocytes were selectively localized to this region, their loss could explain greater injury in polyploid-rich livers. However, prior work shows that polyploid hepatocytes are not exclusively zonated and that LKO mice retain normal hepatic architecture [2, 8, 17, 43, 44]. Thus, the protective phenotype in LKO mice is unlikely to result from altered zonation.

Our results also align with findings in *Mir122* knockout mice, which show increased proportions of diploid hepatocytes (like LKO mice) and APAP resistance [45, 46]. While those effects were attributed to reduced CYP2E1/CYP1A2 expression, diploid enrichment may also contribute. The proliferative advantage of diploid hepatocytes may also support their resistance to APAP injury. Proliferation has been linked to induction of the efflux transporter MRP4, promoting resistance to liver injury after multiple rounds of APAP overdose [47]. These findings suggest that diploid-enriched livers are better equipped for regeneration, detoxification, and stress resilience, supporting a model where increased diploidy enhances liver recovery during acute toxic stress.

Prior studies have suggested that polyploidy enhances cellular functional capacity, potentially through increased gene dosage [48] and modest transcriptional changes. For example, Lu et al. identified small changes in gene expression between ploidy states – such as increased *Nr1i3* and *Ccne2*, and decreased *Gas2* – which may support enhanced stress response [49]. Mietten et al. found that higher ploidy correlates with reduced mitochondrial gene expression, possibly conferring protection from oxidative stress [50]. In contrast, our findings reveal that diploid hepatocytes exhibit superior stress tolerance following APAP injury, mediated by suppressed MKK4 and JNK signaling and enhanced regeneration. Our data also raise the possibility that diploid hepatocytes better preserve mitochondrial integrity under oxidative stress. Although baseline mitochondrial gene expression did not differ between genotypes, injury-induced transcriptional changes in LKO livers point toward enhanced mitochondrial maintenance and redox balance in diploid-enriched tissue. Future work should directly assess mitochondrial function, including ATP synthesis and membrane potential, to determine whether diploid hepatocytes possess an intrinsic bioenergetic advantage that contributes to their resistance to injury.

Collectively, these findings support a model in which hepatocyte ploidy states confer distinct, context-dependent advantages. Polyploid hepatocytes may promote tumor suppression and adaptation to chronic liver injury through mechanisms like metabolic buffering and aneuploidy [1, 6, 13]. In contrast, diploid hepatocytes appear better equipped for acute toxic injury, resisting necrosis and initiating regeneration. This division of labor may allow flexible responses to diverse injury contexts (Fig. 8B). While human data remain limited, studies report wide variability in hepatocyte ploidy among individuals, ranging from 20-50% polyploidy [2, 51–53]. This individual variability raises the possibility that ploidy composition could influence clinical outcomes. We speculate that higher diploid content may confer resilience to APAP toxicity and that hepatocyte ploidy profiling could help predict injury susceptibility.

In conclusion, this study establishes hepatocyte ploidy as a key determinant of susceptibility to APAP-induced liver injury. Diploid hepatocytes resist necrotic cell death through suppression of JNK signaling and initiate robust regeneration, enabling recovery under toxic stress. This work not only defines a mechanistic basis for diploid resilience but also supports a broader model in which ploidy states specify distinct functional roles relevant to human liver disease.

## Materials and methods

### Animals, treatments, and tissue collection

All animal procedures were approved by the University of Pittsburgh Institutional Animal Care and Use Committee and followed NIH guidelines. All experiments were performed exclusively in male mice. WT C57BL/6J mice (Jackson Laboratory, Bar Harbor, ME, #0664) and *E2f7/E2f8* floxed mice (from Drs. Alain de Bruin and Gustavo Leone) [18, 19] were used. Liver-specific *E2f7/E2f8* knockout (LKO) mice were of a mixed genetic background, predominantly FVB with floxed alleles for *E2f7/E2f8*^*loxP/loxP*^, R26R^mTmG/mTmG^, and Alb-Cre^Tg/0^ (Jackson Laboratory, #7676 and #3574) [54, 55]. Control littermates lacked Cre (Alb-*Cre*^*0/0*^). Hepatocyte-specific knockouts (HKO) were generated by intraperitoneal (IP) injection in 2-month-old floxed mice with 1.25 × 10¹¹ AAV8-TBG-Cre viral particles; controls received AAV8-TBG-Null (gifted from James M. Wilson; Addgene, Watertown, MA, 107787-AAV8 and 105536-AAV8).

APAP (16 µg/µL in 0.9% saline) was administered IP at 300 or 600 mg/kg to 2.5-month-old overnight-fasted (~16 h) mice. Livers and blood were collected 0–96 h post-injection. Liver tissue was snap-frozen; serum was isolated by centrifugation (10,000 rpm, 10 min, 4 °C) and analyzed for alanine aminotransferase (ALT) and aspartate aminotransaminase (AST) by the University of Pittsburgh Medical Clinical Laboratory.

Number of animals per group and timepoint: For the 300 mg/kg APAP cohort, control and LKO mice were analyzed at 0 h (*n* = 4 control, 5 LKO), 6 h (*n* = 4 control, 6 LKO), 12 h (*n* = 6 control, 4 LKO), 24 h (*n* = 7 control, 5 LKO), 48 h (*n* = 4 control, 4 LKO), 72 h (*n* = 4 control, 4 LKO), and 96 h (*n* = 3 control, 4 LKO). For the 600 mg/kg “non-regenerating dose” experiment, sample sizes were 0 h (*n* = 4 control, 5 LKO), 6 h (*n* = 5 control, 4 LKO), 12 h (*n* = 4 control, 6 LKO), 24 h (*n* = 10 control, 12 LKO), 48 h (*n* = 4 control, 8 LKO), and 72 h (*n* = 3 control, 8 LKO). In the hepatocyte-specific knockout (HKO) study at 300 mg/kg APAP, animals were analyzed at 0 h (*n* = 4 control, 5 HKO), 12 h (*n* = 5 control, 5 HKO), 24 h (*n* = 6 control, 5 HKO), and 48 h (*n* = 3 control, 6 HKO). Whenever feasible, the same animals were used across multiple endpoints, including serum biochemistry and histology. Minor variation in n between assays reflects sample availability or technical limitations during processing.

### Hepatocyte isolation

Primary hepatocytes were isolated by a two-step collagenase perfusion, as previously published [17].

### In vitro APAP treatment

Primary hepatocytes isolated from adult (2–3 months old; male) WT mice were plated at 3 million cells per 10-cm Primaria plate (Corning, Corning, NY) in seeding medium (DMEM/F-12 with 15 mM HEPES, 5% FBS, and Antibiotic-Antimycotic; Corning). After 4 h, cells were switched to growth medium composed of DMEM/F-12 with 15 mM HEPES, 0.5% FBS, Antibiotic-Antimycotic Solution, and ITS Supplement (1 μg/mL insulin, 0.55 μg/mL transferrin, and 0.67 ng/mL sodium selenite; ThermoFisher). Cells were allowed to adhere overnight. The following morning, cells were treated with 0–10 mM APAP diluted in growth medium. After 24 h, cells were imaged, trypsinized, and stained with FVD780 and Hoechst for flow cytometric analysis of viability and ploidy.

### Ploidy analysis

Hepatocyte ploidy was evaluated by flow cytometry as previously described [15], using an LSR II flow cytometer (BD Biosciences, Franklin Lakes, NJ) operated with BD FACSDiva™ Software v9.0. Data were analyzed and FACS plots generated using FlowJo v10.8.2 (FlowJo LLC, Ashland, OR).

### Protein isolation and Western blotting

Lysates were prepared in T-PER buffer (ThermoFisher, Waltham, MA) with Halt protease/phosphatase inhibitors (ThermoFisher) and homogenized. Protein was quantified (Pierce PCA Assay; ThermoFisher), separated by SDS-PAGE (4–12% Bis-Tris gels), transferred to PVDF membranes (Millipore, Burlington, MA), blocked (Pierce Protein-Free buffer, ThermoFisher), and probed with primary and secondary antibodies (Supplementary Materials and Methods). Detection used SuperSignal Pico substrate (ThermoFisher) and ChemiDoc Touch (Bio-Rad, Hercules, CA). Full, uncropped western blots are included as a Supplementary file.

### Histology, immunohistochemistry, and TUNEL staining

Liver tissue was fixed in 10% neutral-buffered formalin, paraffin-embedded, and sectioned at 4μm. Hematoxylin and eosin (H&E) staining was performed by the Pitt Biospecimen Core Research Histology Lab. Nitrotyrosine immunohistochemistry was performed on liver sections using an anti-nitrotyrosine primary antibody (Invitrogen A21285) with DAB chromogenic detection. DNA fragmentation was assessed using the Click-iT® Plus TUNEL Assay Kit (ThermoFisher) according to the manufacturer’s protocol, and quantification was performed on liver sections centered on the central vein, where TUNEL-positive and -negative nuclei were manually counted across the full field.

### Glutathione measurement

Total hepatic glutathione (GSH) was measured in liver homogenates using the Glutathione Colorimetric Detection Kit (Invitrogen, Carlsbad, CA) per the manufacturer’s instructions.

### RNA sequencing and analysis

Total RNA was extracted from snap-frozen liver tissue using TRIzol reagent (Life Technologies, Carlsbad, CA). For baseline (0 h) samples, RNA was isolated from individual mice (*n* = 4 per genotype). For 12–96 h time points, RNA from three mice per genotype per time point was pooled. All samples were submitted to Novogene (Sacramento, CA) for quality control, library preparation, and sequencing. Adapter sequences and low-quality reads were removed using Trimmomatic (v0.38) [56]. Clean reads were aligned to the Mus musculus reference genome (mm10) using STAR aligner (v2.6.1a) [57], and gene-level read counts were quantified with --quantMode GeneCounts option. Differential gene expression was assessed using DESeq2, with a false discovery rate (FDR) of 0.05 and a minimum fold change of 1.5. Canonical pathway and upstream regulator analyses were conducted using Ingenuity Pathway Analysis (IPA; Qiagen, Hilden, Germany, version 01-10). Genes with similar temporal expression profiles were clustered using tight clustering [58]. Raw RNA-seq data were submitted to the Gene Expression Omnibus database (http://www.ncbi.nlm.nih.gov/geo; accession number GSE303454).

### Microscopy

Micrographs were captured with a TiU fluorescent microscope (Nikon, Melville, NY) equipped with a Moment sCMOS monochrome camera (fluorescent images) (Photometrics, Tucson, AZ) or Nikon DS-Fi3 color camera (non-fluorescent images). The display lookup table is linear and covers the full range of the data. Images were processed with Nikon NIS Elements Advanced Research software and QuPath v0.4.2 [59].

### Statistical analysis

Data are mean ± SEM. GraphPad Prism 10 was used. Two-group comparisons used unpaired two-tailed t-tests. Outliers were identified using Grubbs’ test (*α* = 0.01). Significance was set at *P* < 0.05. The sample size used for all studies, including animal studies, was based on prior experience [15, 17, 29]. LKO and control mice were assigned to treatment groups by simple randomization; analyses were performed in a blinded fashion.

## Supplementary information

Supplementary Information

Uncropped Western Blots

Supplementary Tables

## Data Availability

Genomic data are deposited in the Gene Expression Omnibus database (http://www.ncbi.nlm.nih.gov/geo; accession number GSE303454). Additional data supporting the findings of this study are available from the corresponding author upon reasonable request.
