# Supplementary material for: Diploid hepatocytes resist acetaminophen-induced liver injury through suppressed JNK signaling
Source: Cell Death Dis. 2026 Feb 3;17(1):203. doi: 10.1038/s41419-026-08448-z (PMC12894925; doi:10.1038/s41419-026-08448-z)
Supplement: Supplementary file 1 — Supplementary Information [file 41419_2026_8448_MOESM1_ESM.pdf]

Diploid hepatocytes resist acetaminophen-induced liver injury through suppressed JNK signaling

Sierra R. Wilson, Evan R. Delgado, Rosa L. Loewenstein, Frances Alencastro, Madeleine P. Leek, Leah R. Peters, Siddhi Jain, Kerollos Kamel, Patrick D. Wilkinson, Silvia Liu, Joseph Locker, Bharat Bhushan, Andrew W. Duncan

Supplementary Information

Supplementary Materials and Methods

Antibodies Used for Western Blotting

Supplementary Figures

Supplementary Fig. S1. Cell size and ploidy analysis in control and LKO hepatocytes.

Supplementary Fig. S2. LKO mice resist lethal dose of APAP (600 mg/kg).

Supplementary Fig. S3. LKO mice show enhanced proliferation at 48 hours.

Supplementary Fig. S4. Expression in the HKO model.

Supplementary Fig. S5. Sirtuin expression in LKO livers.

Supplementary Fig. S6. JNK activation following APAP overdose in LKO and HKO mice.

Supplementary Materials and Methods

Antibodies Used for Western Blotting

Primary Antibodies

Epitope	*Antibody with species reactivity*	*Vendor*	*Catalog #*
AIF	Rabbit anti-AIF	Cell Signaling	4642
Cyclin D1	Rabbit anti-Cyclin D1	Cell Signaling	55506
CYP1A2	Goat anti-CYP1A2	Santa Cruz	sc-9835
CYP2E1	Rabbit anti-CYP2E1	MilliporeSigma	ab1252
GAPDH	Rabbit anti-GAPDH	Cell Signaling	5174
SEK1/MKK4	Rabbit anti-SEK1/MKK4	Cell Signaling	9152
Non-phospho β-CATENIN	Rabbit anti-Non-phospho β-CATENIN	Cell Signaling	19807
PCNA	Mouse anti-PCNA	Cell Signaling	2586
Phospho-SEK1/MKK4	Rabbit anti-Phospho-SEK1/MKK4	Cell Signaling	9155
Phospho-SAPK/JNK	Rabbit anti-Phospho-SAPK/JNK	Cell Signaling	4668
Phospho-β-CATENIN	Rabbit anti-Phospho-β-CATENIN	Cell Signaling	9561
SAPK/JNK	Rabbit anti-SAPK/JNK	Cell Signaling	9252
SIRT1	Rabbit anti-SIRT1	Cell Signaling	3931
SIRT3	Rabbit anti-SIRT3	MilliporeSigma	07-1596
SIRT6	Rabbit anti-SIRT6	Cell Signaling	12486
VDAC	Rabbit anti-VDAC	Cell Signaling	4661
β-ACTIN	Rabbit anti-β-ACTIN	Cell Signaling	4970
β-CATENIN	Rabbit anti-β-CATENIN	Cell Signaling	8480

Secondary Antibodies

Species reactivity	*Antibody with conjugate*	*Vendor*	*Catalog #*
Mouse	Anti-mouse IgG	Cell Signaling	7076
Rabbit	Anti-rabbit IgG	Cell Signaling	7074

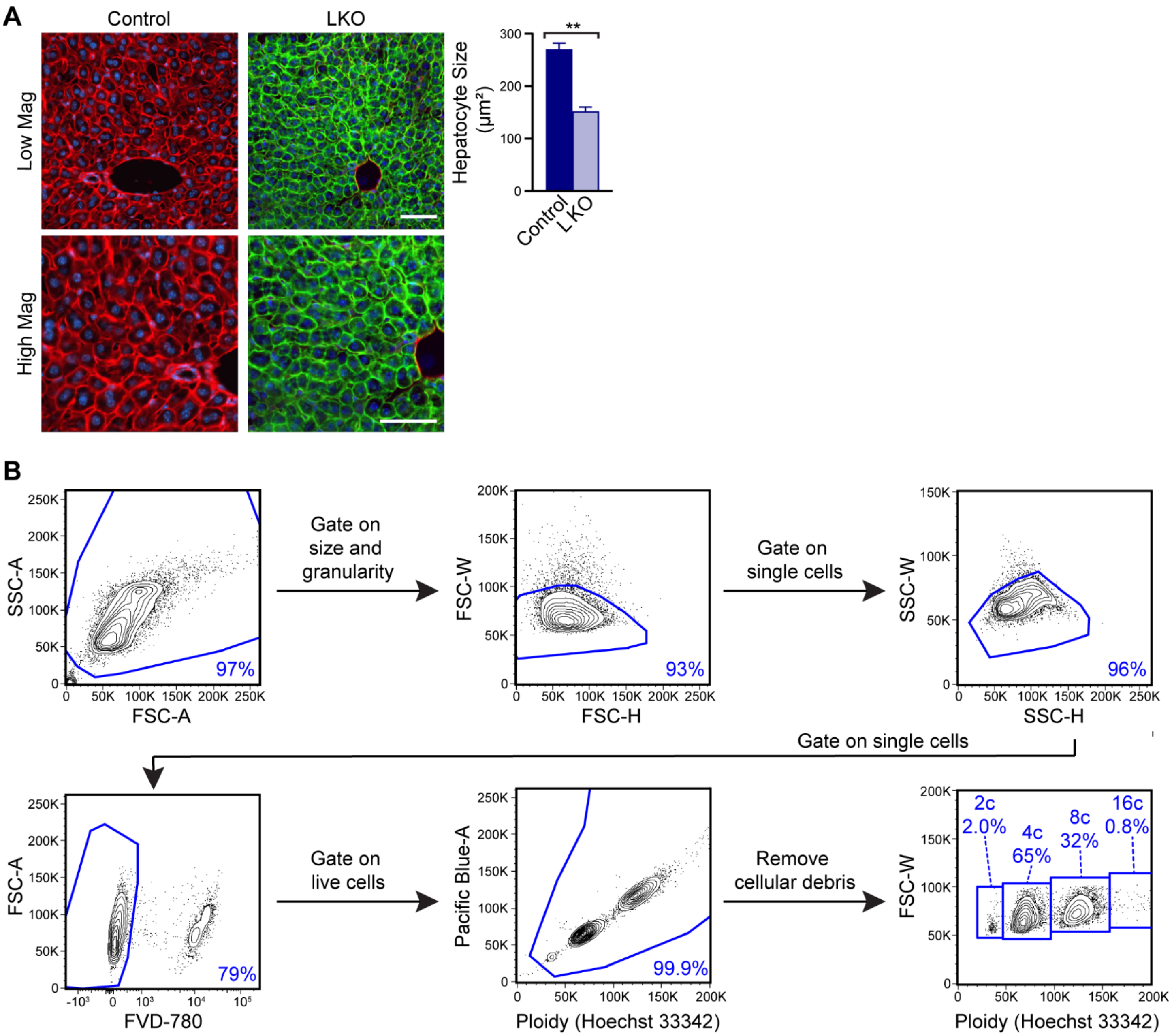

Supplementary Fig. S1. Cell size and ploidy analysis in control and LKO hepatocytes. (A) Representative lower and higher magnification images showing mTmG reporter expression (tdTomato, red; GFP, green) and nuclear staining with Hoechst 33342 (blue). In the R26R-mTmG Cre-reporter system, all cells express membrane-bound tdTomato (red) prior to Cre recombination. Upon Cre-mediated excision, tdTomato is replaced by membrane-bound GFP (green), indicating successful recombination. Scale bars = 50 μm. Quantification of average hepatocyte cell area (μm²) is shown (n = 3/genotype). **(B)** Flow cytometry gating strategy for ploidy analysis. Single-cell suspensions of hepatocytes from control and LKO mice were stained with FVD780 (fixable viability dye) and Hoechst. Cells were first gated based on size and granularity, then on single cells, followed by exclusion of dead cells and debris. Ploidy populations were determined by Hoechst 33342 fluorescence intensity. Shown plots are representative of the control mouse in Fig. 1B. Graphs show mean ± SEM. *P < 0.05; **P < 0.01; ***P < 0.001.

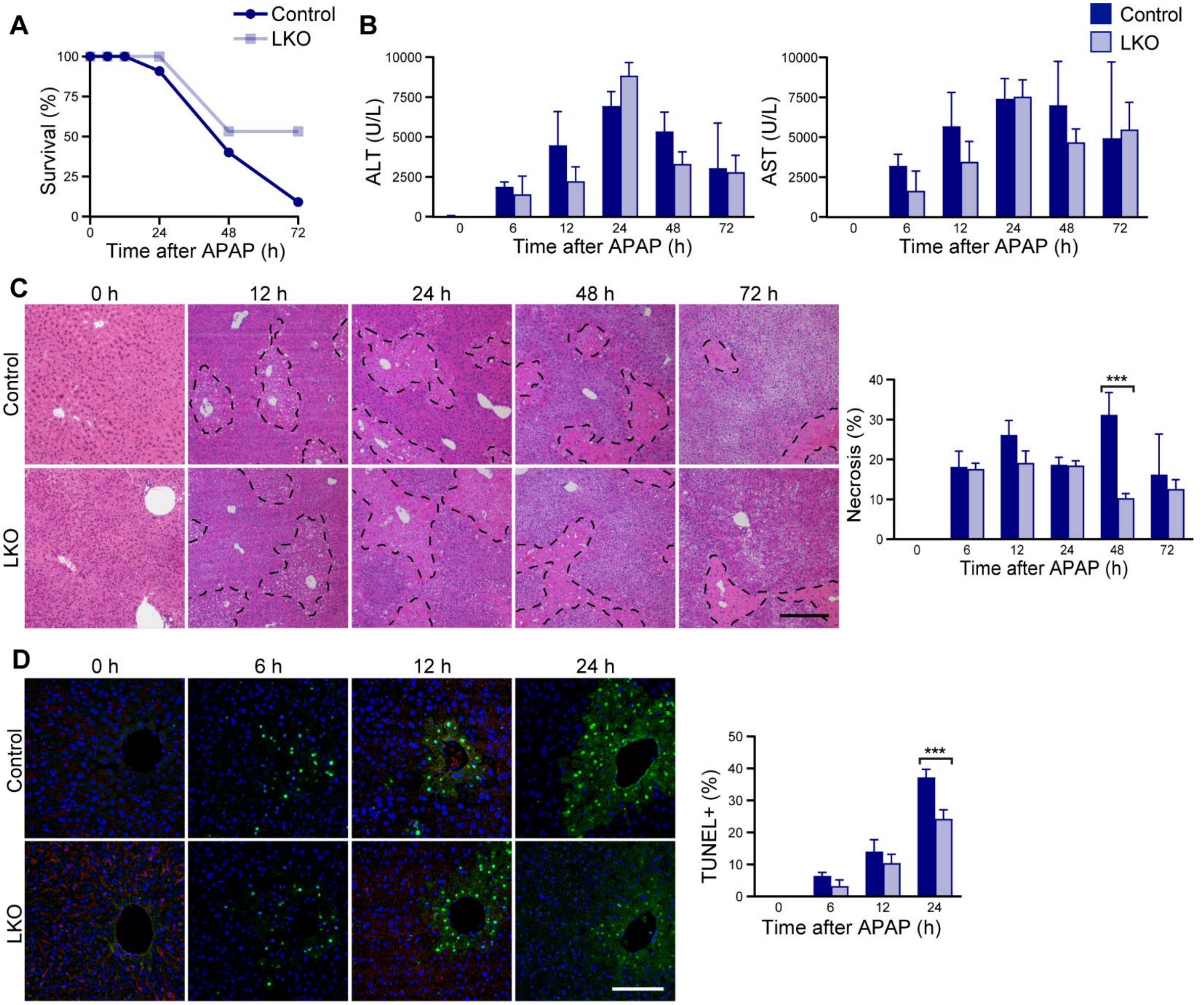

Supplementary Fig. S2. LKO mice resist lethal dose of APAP (600 mg/kg). (A) Survival curves of control and LKO mice following 600 mg/kg APAP overdose (n = 4-12/genotype/timepoint). **(B)** Levels of liver biomarkers ALT and AST in the serum (n = 4-12/genotype/timepoint). **(C)** Quantification of necrosis by H&E Staining (n = 4-12/genotype/timepoint). Scale bar = 200 µm. **(D)** TUNEL staining showing DNA fragmentation (green) and nuclear staining with Hoechst 33342 (blue) (n = 4-9/genotype/timepoint). Scale bar = 100 µm. Representative images, plots, and quantification results are shown. Graphs show mean ± SEM. *P < 0.05; **P < 0.01; ***P < 0.001.

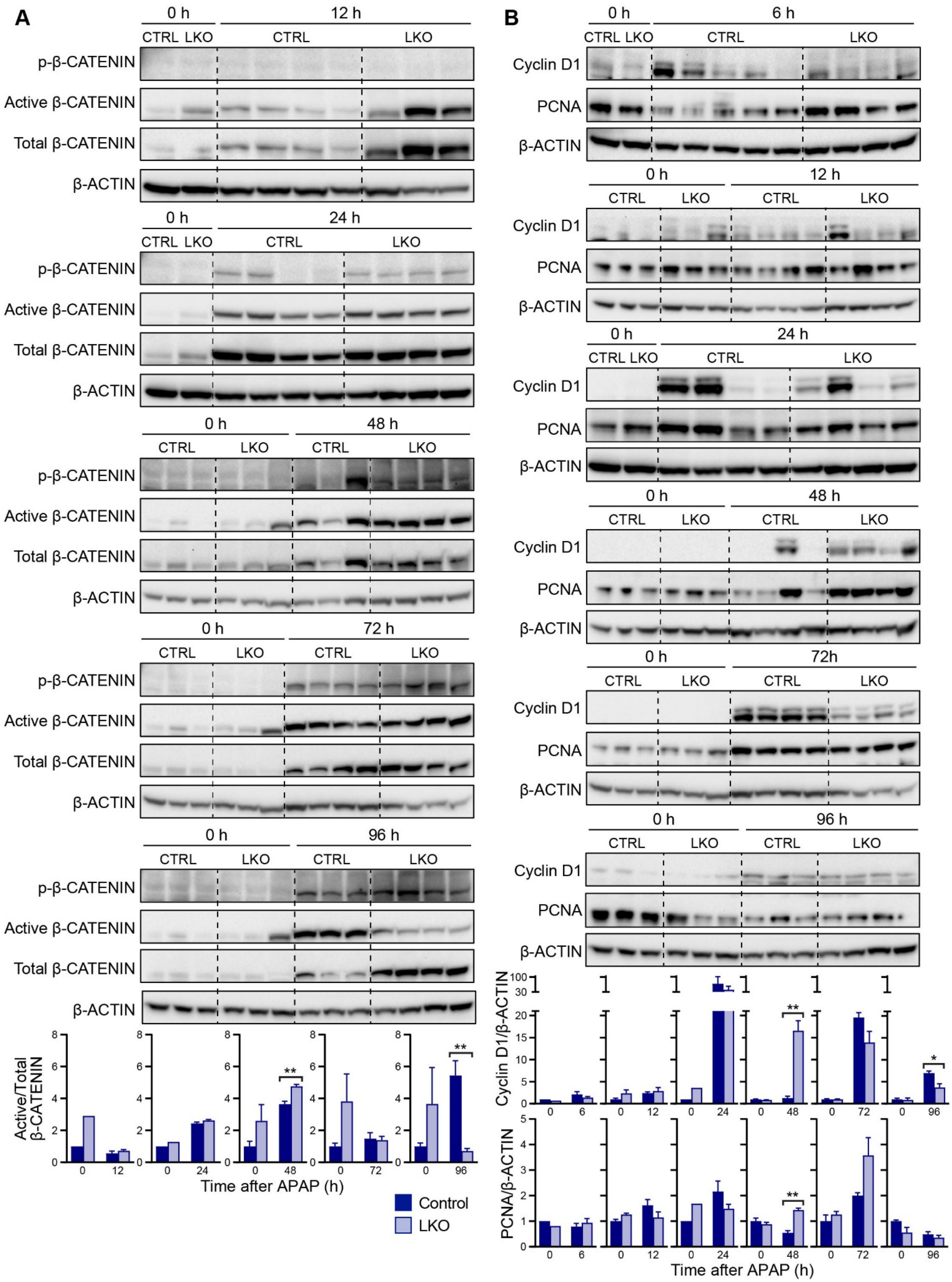
A
B
0 h
12 h
24 h
48 h
72 h
96 h
6 h
CTRL
LKO
p-β-CATENIN
Active β-CATENIN
Total β-CATENIN
β-ACTIN
Cyclin D1
PCNA
Active/Total β-CATENIN
Cyclin D1/β-ACTIN
PCNA/β-ACTIN
Time after APAP (h)
Control
LKO
**
*

Supplementary Fig. S3. LKO mice show enhanced proliferation at 48 hours. (A-B) Western blotting of whole liver lysates collected from control and LKO mice 0-96 hours after 300 mg/kg APAP overdose, showing expression of proteins involved in proliferation: **(A)** active β-CATENIN relative to total β-CATENIN (n = 3-4/genotype/timepoint), **(B)** Cyclin D1 and PCNA relative to β-ACTIN where one sample at 48 hours was identified as an outlier using the Grubbs Test with an alpha = 0.01 per treatment group (n = 3-5/genotype/timepoint). Shown are representative blots and quantification results normalized to the 0 hour control, which is set to 1. Graphs show mean ± SEM. *P < 0.05; **P < 0.01; ***P < 0.001.

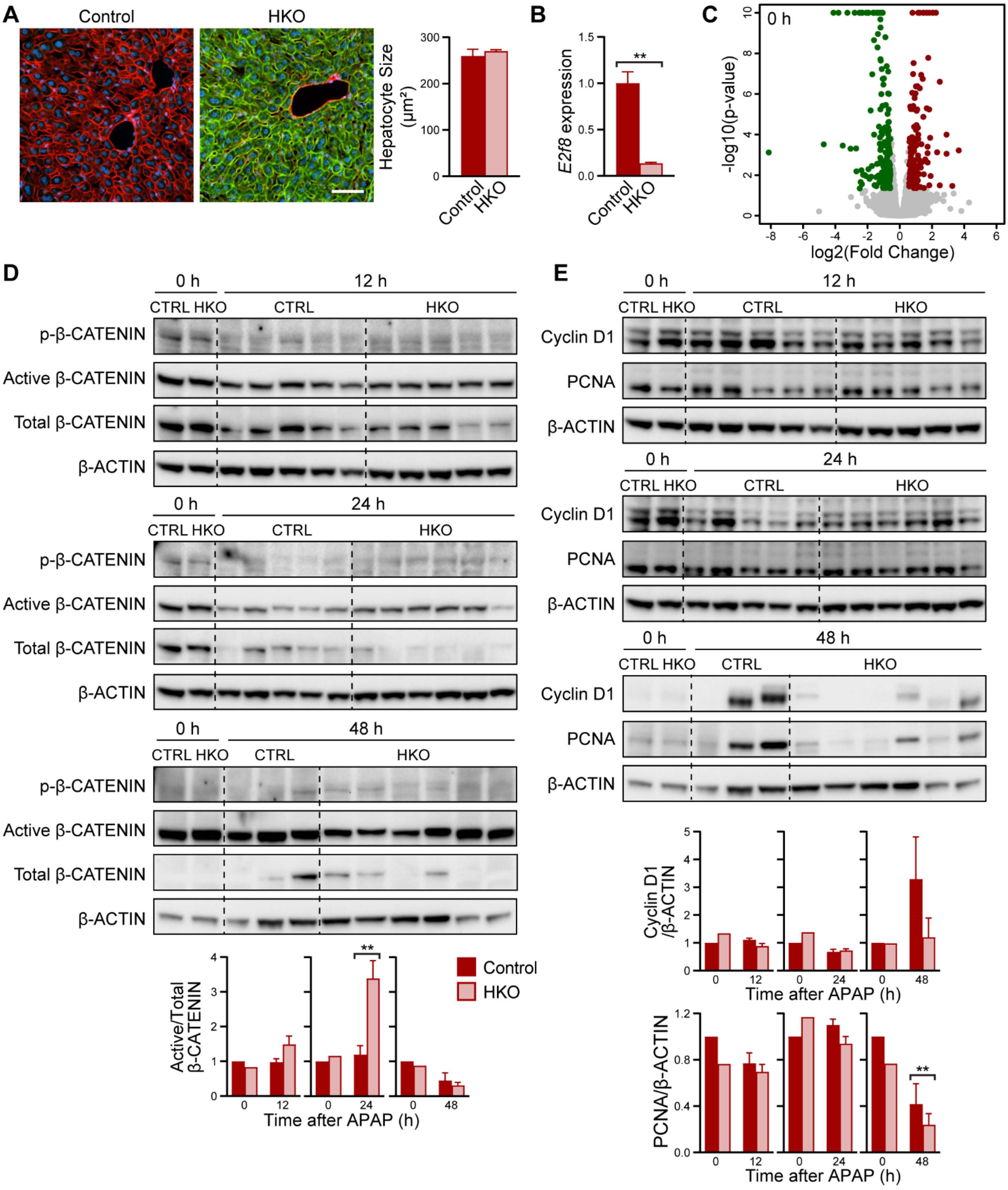

Supplementary Fig. S4. Expression in the HKO model. (A) Representative fluorescent images of liver sections from control and HKO mice two weeks after AAV8 injection. In the R26R-mTmG Cre-reporter system, all cells express membrane-bound tdTomato (red) prior to Cre recombination. Upon Cre-mediated excision,

tdTomato is replaced by membrane-bound GFP (green), indicating successful recombination. All hepatocytes in control livers remained $tdTomato^{+}$, while >99% of hepatocytes in HKO livers were GFP^{+}, demonstrating efficient, hepatocyte-specific Cre recombinase activity. Scale bar = 50 μm. Quantification of average hepatocyte cell area (μm^2) is shown (n = 3/genotype). **(B)** Expression of *E2f8* in control and HKO livers, based on RNA-seq gene counts in exons 3 and 4. Quantification is normalized to control, which is set to 1. **(C)** Volcano plot showing differential gene expression between control and HKO mice. Differentially expressed genes were defined by FDR = 5% and fold change ≥ 1.5. A total of 141 genes were upregulated (red) and 179 downregulated (green) in HKO compared to controls (Supplementary Table S1). **(D-E)** Western blotting of whole liver lysates collected from control and HKO mice 12, 24, and 48 hours after APAP overdose, showing expression of proteins involved in proliferation **(C)** active β-CATENIN relative to total β-CATENIN and **(D)** CCND1 and PCNA relative to β-ACTIN. Shown are representative blots and quantification results normalized to the 0 hour control, which is set to 1. Graphs show mean ± SEM. *P < 0.05; ** P < 0.01; ***P < 0.001.

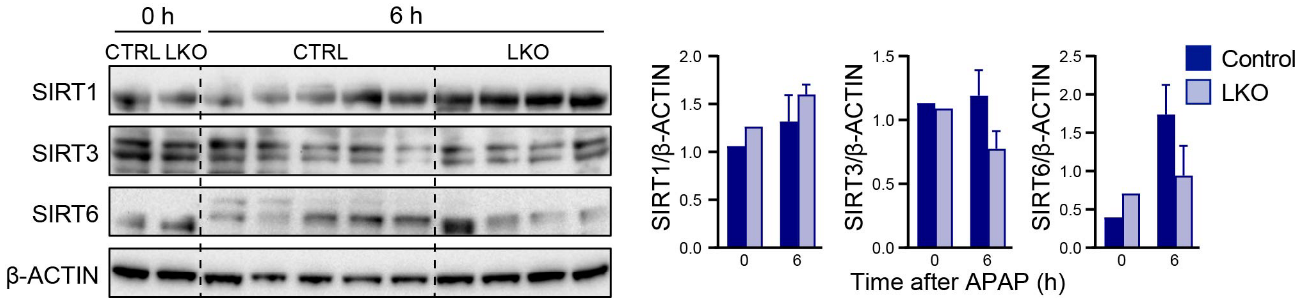

Supplementary Fig. S5. Sirtuin expression in LKO livers. Western blot analysis of SIRT1, SIRT3, and SIRT6 protein levels at 0 and 6 hours post-APAP treatment. Representative blots and quantification are shown. Data are normalized to control at baseline and expressed as mean ± SEM.

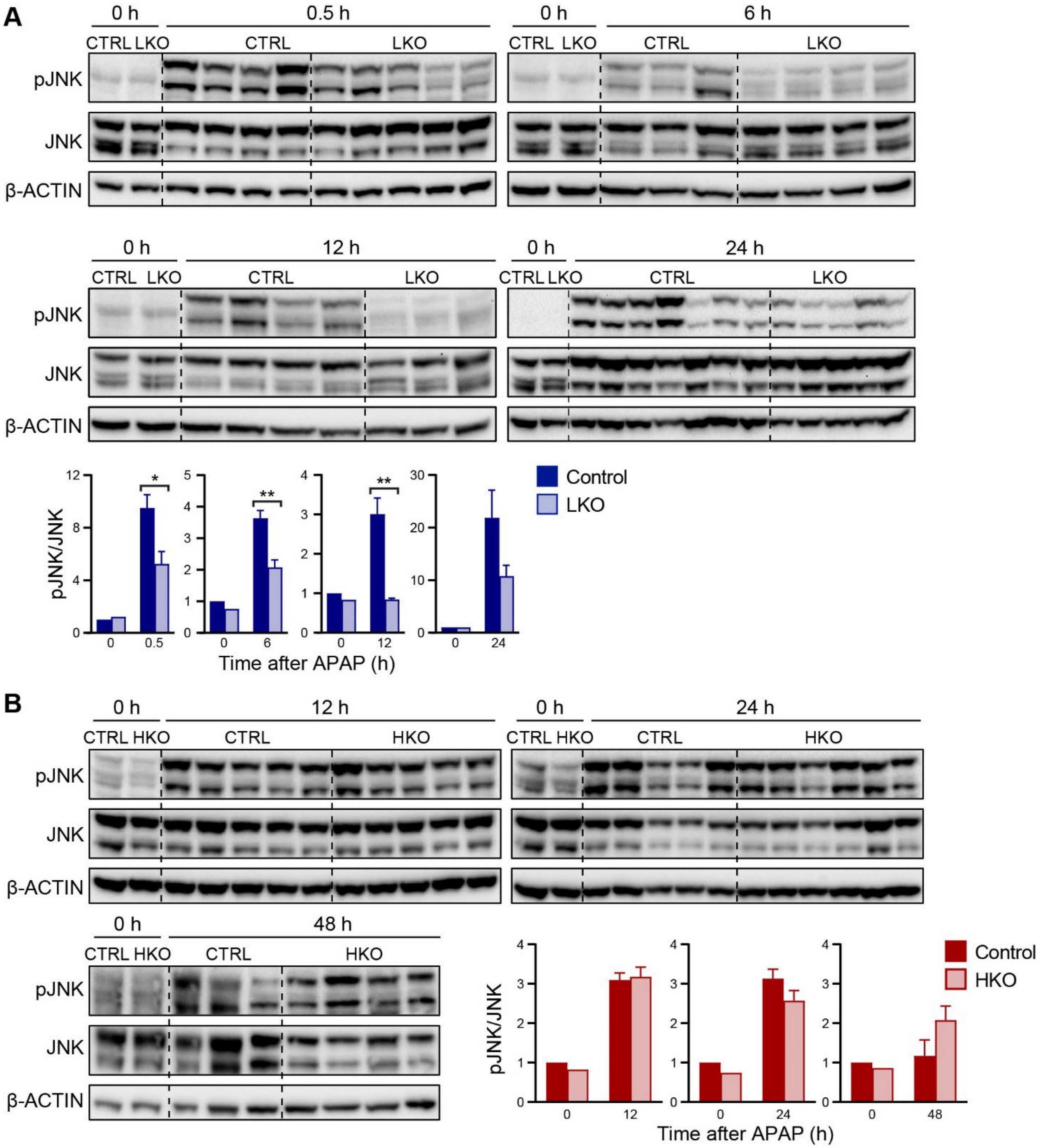

Supplementary Fig. S6. JNK activation following APAP overdose in LKO and HKO mice. (A-B) Western blotting of phosphorylated JNK (pJNK) relative to total JNK in whole-liver lysates after 300 mg/kg APAP. **(A)** Control vs LKO mice over 0-24 hours post-APAP (n = 3-6/genotype/timepoint). **(B)** Control and HKO mice 6 hours after APAP (n = 3-4/genotype). Representative blots and quantification are shown. Data are normalized to control at baseline and expressed as mean ± SEM. *P < 0.05; **P < 0.01; ***P < 0.001.

References

1. Bolger AM, Lohse M, Usadel B. Trimmomatic: a flexible trimmer for Illumina sequence data. *Bioinformatics*. Aug 1 2014;30(15):2114-20.
2. Dobin A, Davis CA, Schlesinger F, et al. STAR: ultrafast universal RNA-seq aligner. *Bioinformatics*. Jan 1 2013;29(1):15-21.
3. Tseng GC, Wong WH. Tight clustering: a resampling-based approach for identifying stable and tight patterns in data. *Biometrics*. Mar 2005;61(1):10-6.
4. Bankhead P, Loughrey MB, Fernández JA, et al. QuPath: Open source software for digital pathology image analysis. *Sci Rep*. Dec 4 2017;7(1):16878.
