# Supplementary material for: Diploid hepatocytes resist acetaminophen-induced liver injury through suppressed JNK signaling
Source: Cell Death Dis. 2026 Feb 3;17(1):203. doi: 10.1038/s41419-026-08448-z (PMC12894925; doi:10.1038/s41419-026-08448-z)
Supplement: Supplementary file 2 — Uncropped Western Blots [file 41419_2026_8448_MOESM2_ESM.pdf]

Diploid Hepatocytes Resist Acetaminophen-Induced Liver Injury Through Suppressed JNK Signaling

Sierra R. Wilson, Evan R. Delgado, Rosa L. Loewenstein, Frances Alencastro, Madeleine P. Leek, Leah R. Peters, Siddhi Jain, Kerollos Kamel, Patrick D. Wilkinson, Silvia Liu, Joseph Locker, Bharat Bhushan, Andrew W. Duncan

Uncropped Western Blots

Uncropped Western Blots and Corresponding Figure

- **A.** Uncropped Western Blots for Fig. 2A
- **B.** Uncropped Western Blots for Fig. 2B
- **C.** Uncropped Western Blots for Fig. 2C
- **D.** Uncropped Western Blots for Fig. 4B
- **E.** Uncropped Western Blots for Fig. 4D
- **F.** Uncropped Western Blots for Fig. 6A
- **G.** Uncropped Western Blots for Fig. 6B
- **H.** Uncropped Western Blots for Fig. 6C
- **I.** Uncropped Western Blots for Fig. 6E
- **J.** Uncropped Western Blots for Supplementary Fig. S3A
- **K.** Uncropped Western Blots for Supplementary Fig. S3B
- **L.** Uncropped Western Blots for Supplementary Fig. S4D
- **M.** Uncropped Western Blots for Supplementary Fig. S4E
- **N.** Uncropped Western Blots for Supplementary Fig. S5
- **O.** Uncropped Western Blots for Supplementary Fig. S6A
- **P.** Uncropped Western Blots for Supplementary Fig. S6B

A. Uncropped Western Blots for Fig. 2A

92 -
kDa

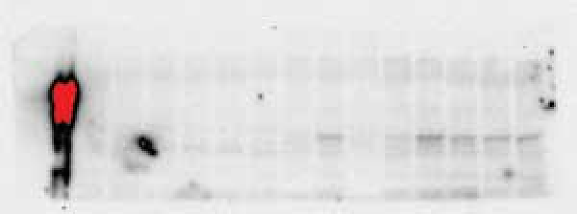

p-β-CATENIN

92 -
kDa

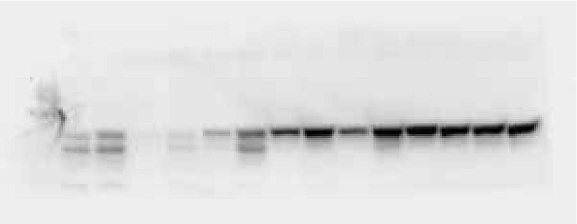

Total β-CATENIN

92 -
kDa

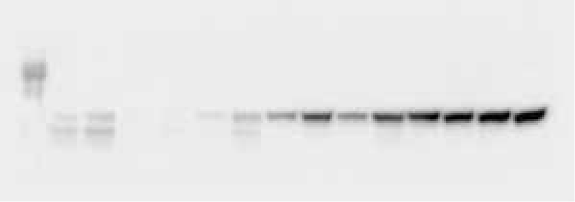

Active β-CATENIN

45 -
kDa

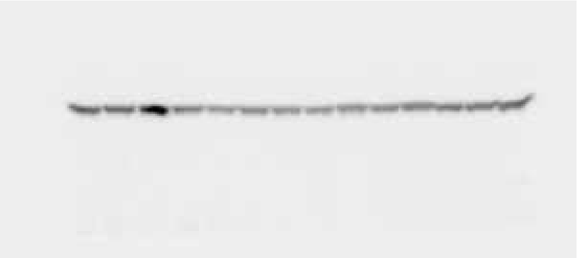

β-ACTIN

B. Uncropped Western Blots for Fig. 2B

38 -
36 -
kDa

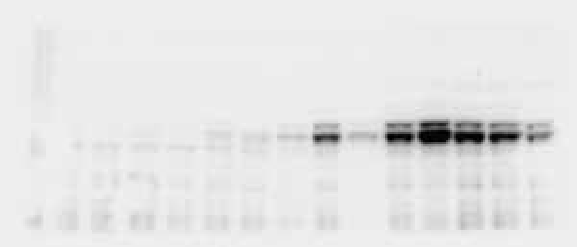

Cyclin D1

45 -
kDa

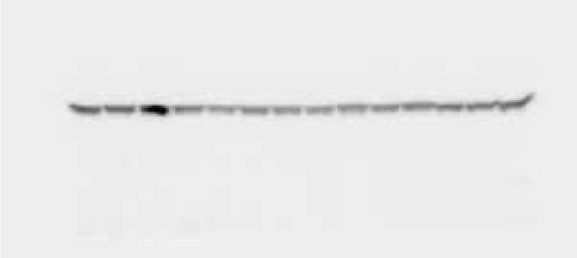

β-ACTIN

C. Uncropped Western Blots for Fig. 2C

36 -
kDA

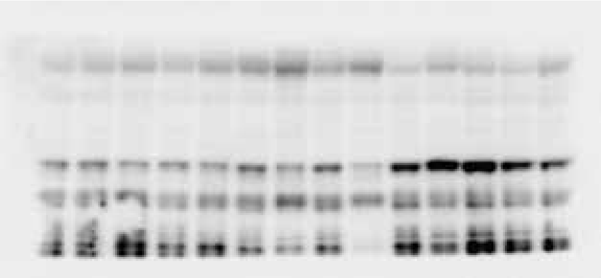

PCNA

45 -
kDa

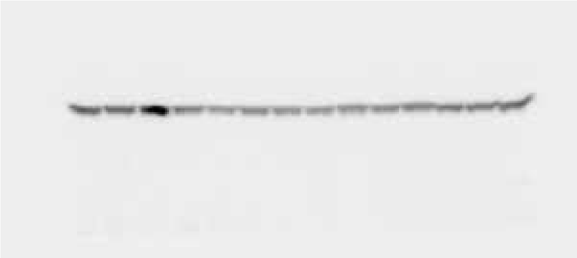

β-ACTIN

D. Uncropped Western Blots for Fig. 4B

54 -
kDa

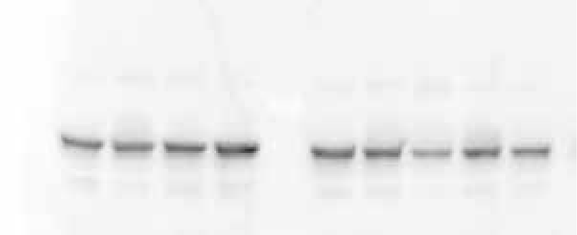

CYP1A2

57 -
kDa

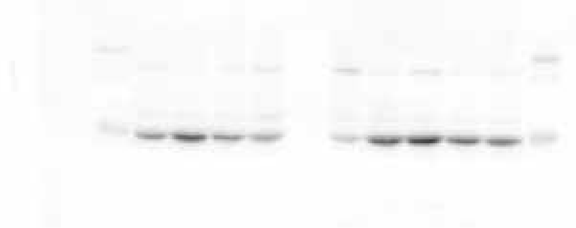

CYP1A2

37 -
kDa

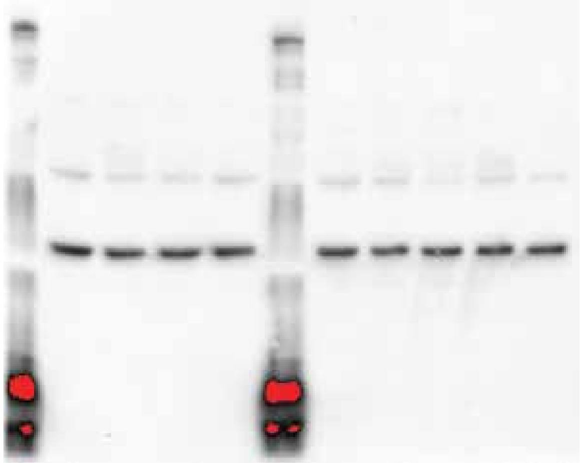

GAPDH

37 -
kDa

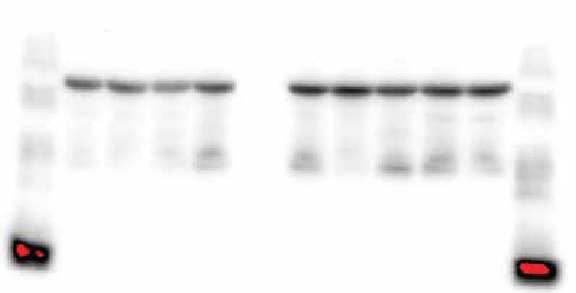

GAPDH

E. Uncropped Western Blots for Fig. 4D

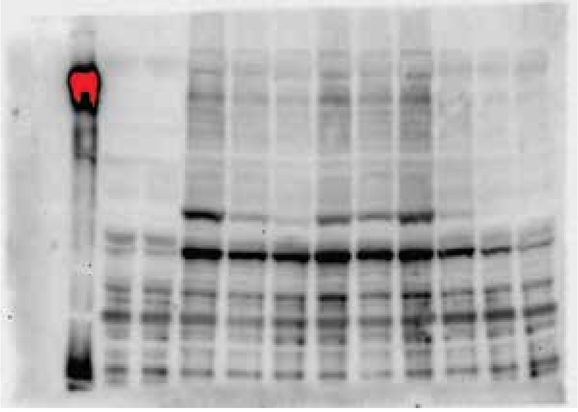

APAP Adducts 0.5 hours

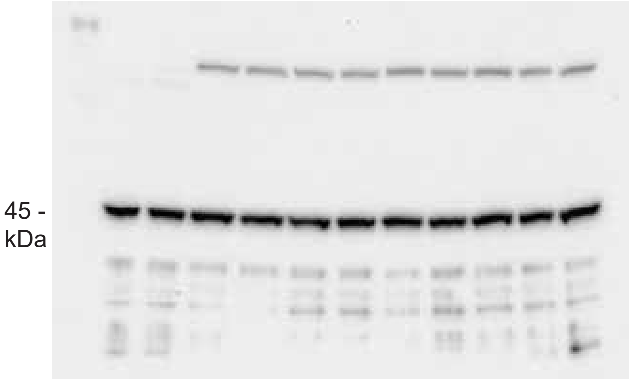

β-ACTIN 0.5 hours

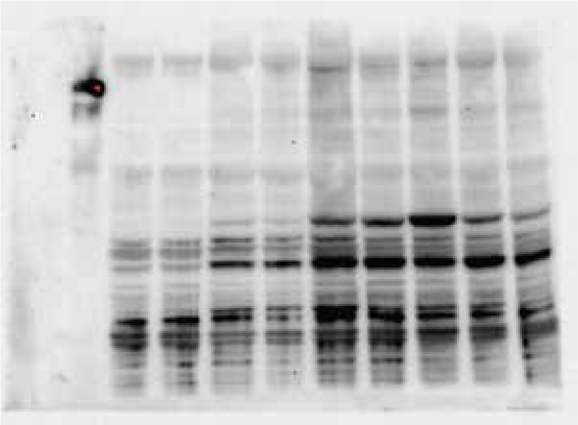

APAP Adducts 6 hours

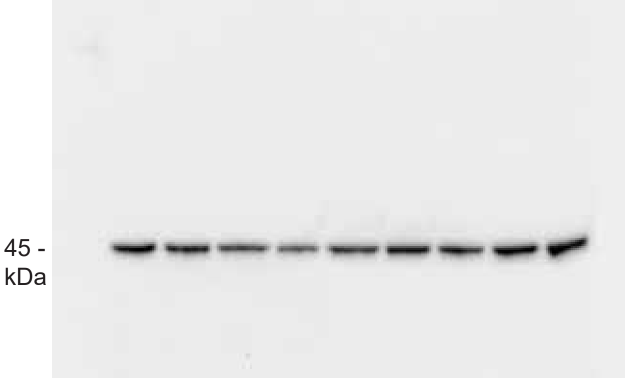

β-ACTIN 6 hours

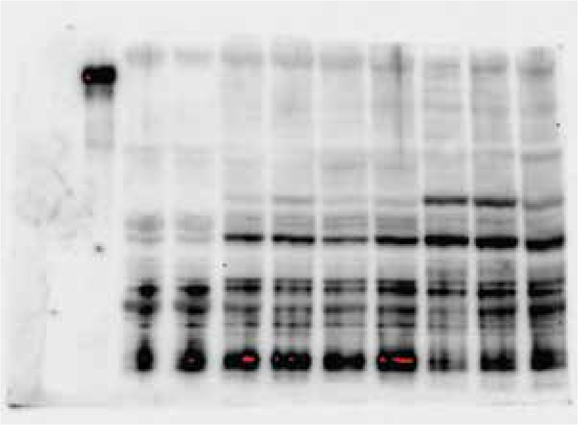

APAP Adducts 12 hours

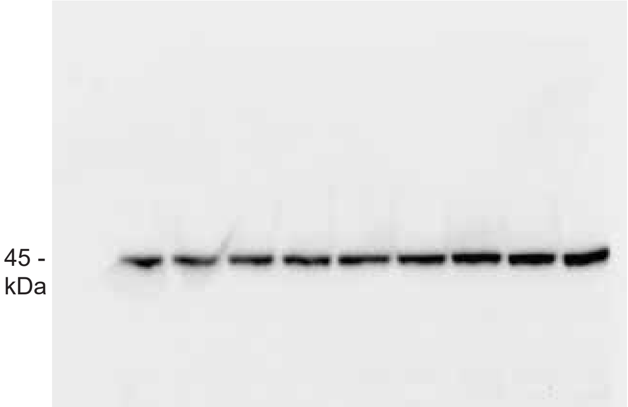

β-ACTIN 12 hours

F. Uncropped Western Blots for Fig. 6A

44 - kDa

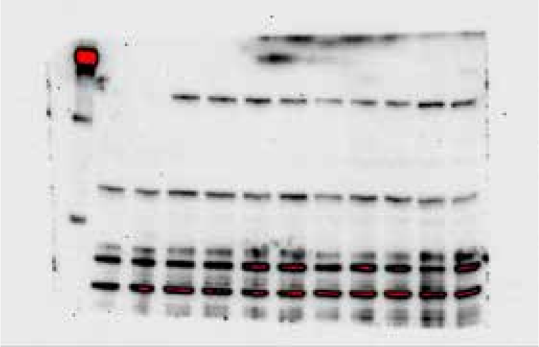

pMKK4 0.5 hours

44 - kDa

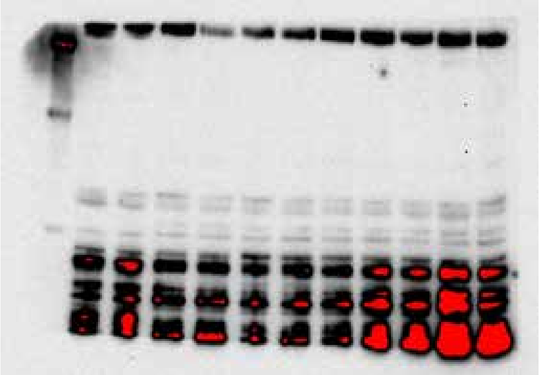

Total MKK4 0.5 hours

45 - kDa

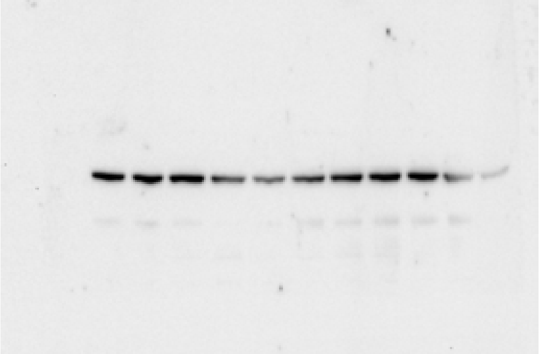

β-ACTIN 0.5 hours

44 - kDa

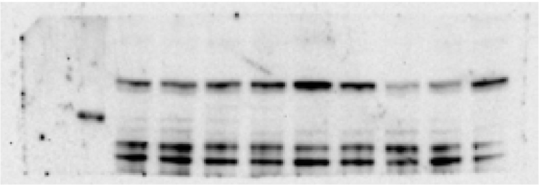

pMKK4 12 hours

44 - kDa

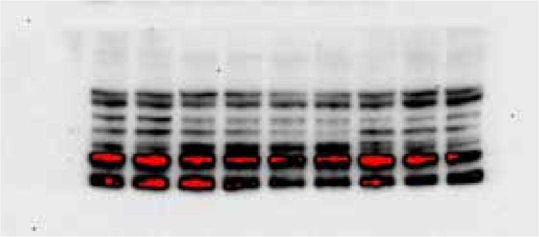

Total MKK4 12 hours

44 - kDa

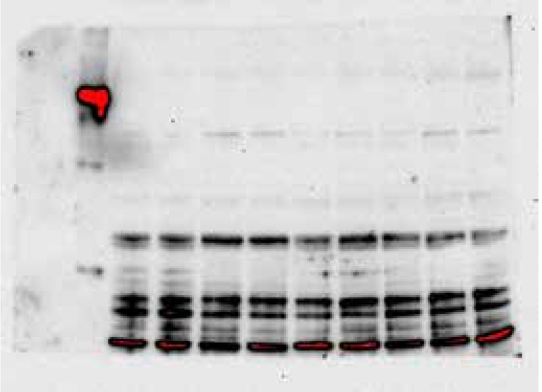

pMKK4 6 hours

44 - kDa

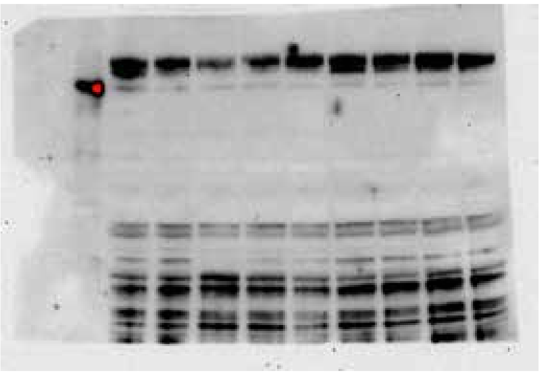

Total MKK4 6 hours

45 - kDa

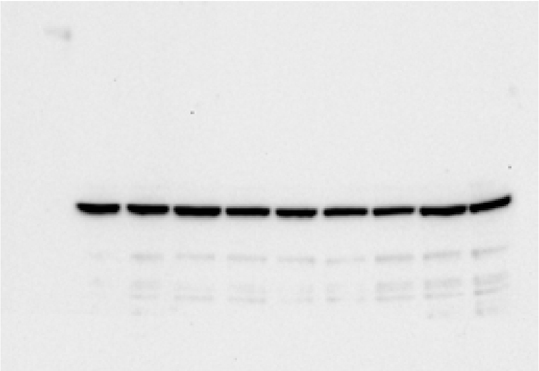

β-ACTIN 6 hours

45 - kDa

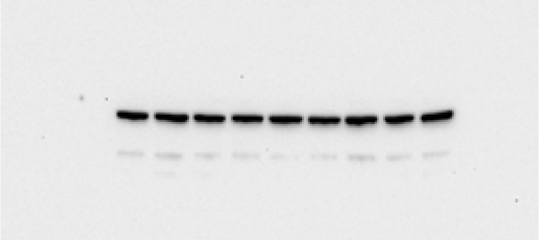

β-ACTIN 12 hours

G. Uncropped Western Blots for Fig. 6B

54 -
46 -
kDa

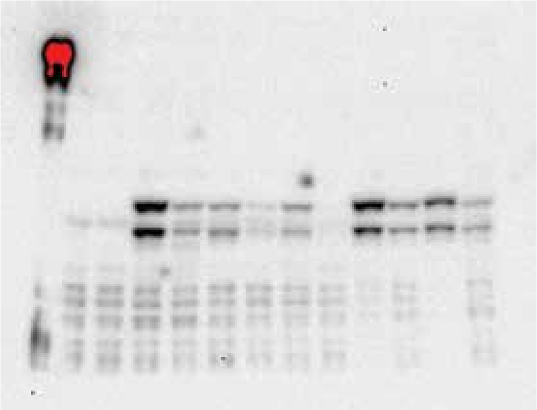

pJNK

54 -
46 -
kDa

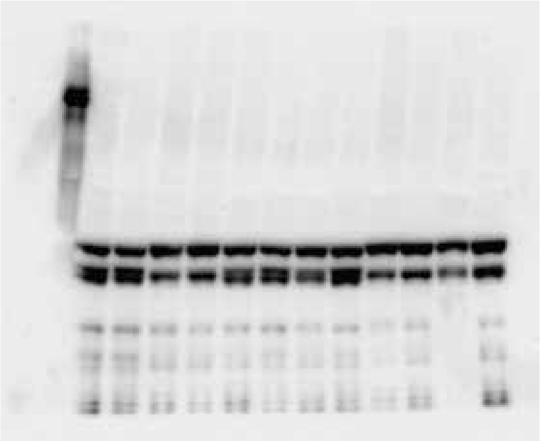

JNK

45 -
kDa

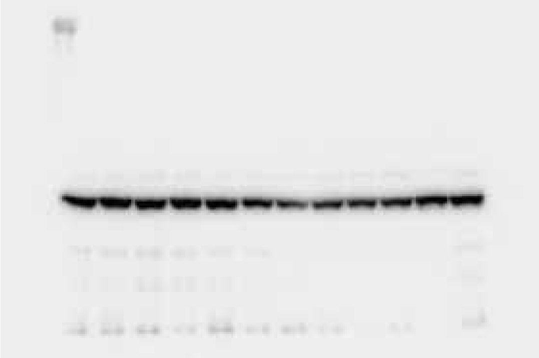

β-ACTIN

H. Uncropped Western Blots for Fig. 6C

54 -
46 -
kDa

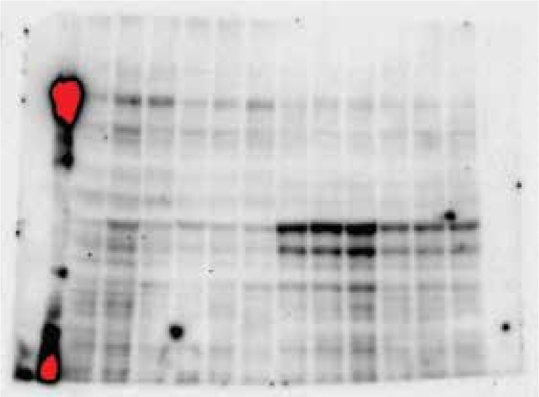

pJNK

54 -
46 -
kDa

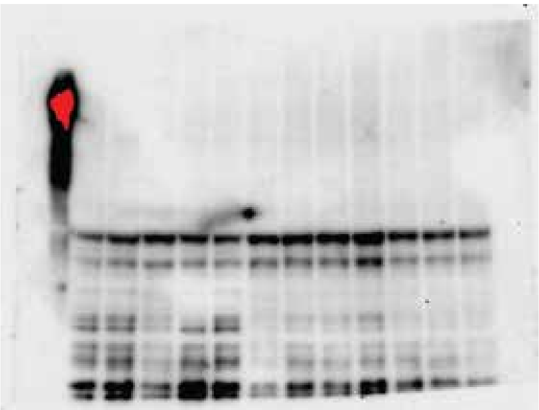

JNK

32 -
kDa

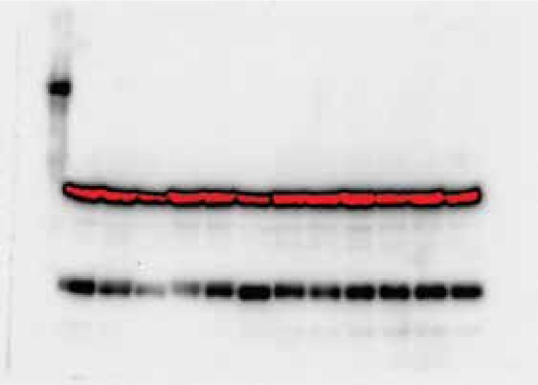

VDAC

I. Uncropped Western Blots for Fig. 6E

57 -
kDa

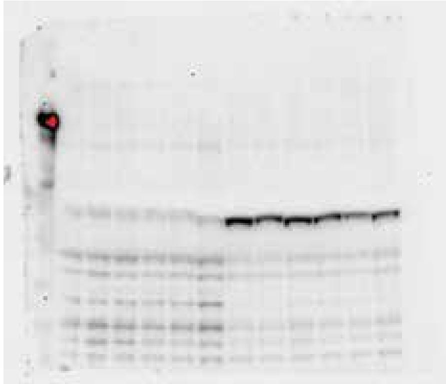

AIF

37 -
kDa

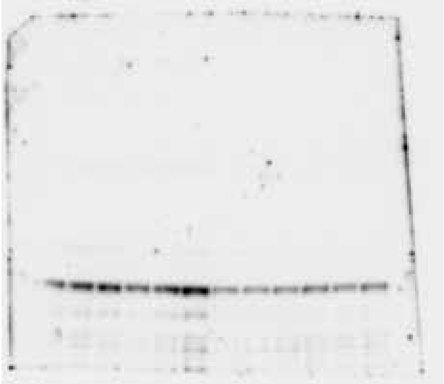

GAPDH

J. Uncropped Western Blots for Supplementary Fig. S3A

92 - kDa

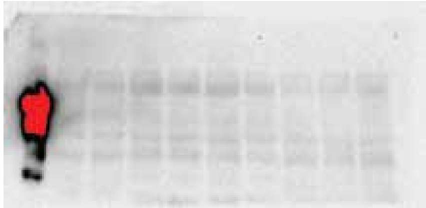

p-β-CATENIN 12 hours

92 - kDa

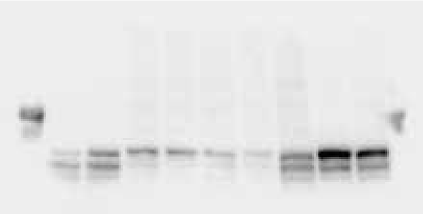

Active β-CATENIN 12 hours

92 - kDa

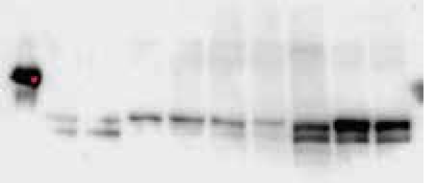

Total β-CATENIN 12 hours

45 - kDa

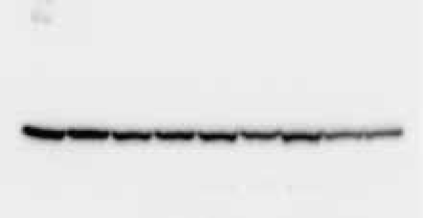

β-ACTIN 12 hours

92 - kDa

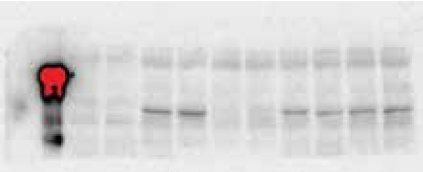

p-β-CATENIN 24 hours

92 - kDa

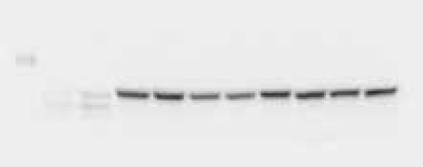

Active β-CATENIN 24 hours

92 - kDa

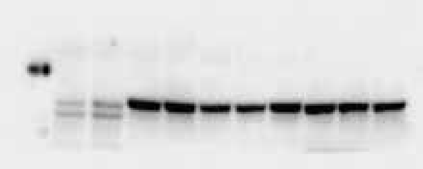

Total β-CATENIN 24 hours

45 - kDa

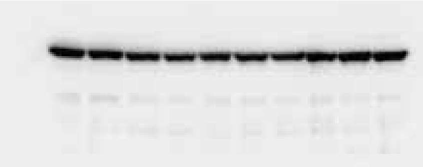

β-ACTIN 24 hours

92 - kDa

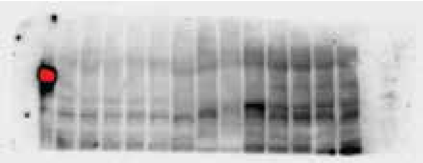

p-β-CATENIN 48 hours

92 - kDa

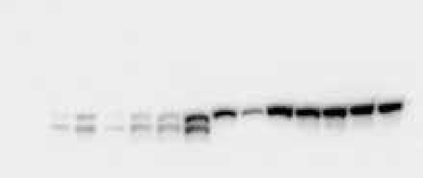

Active β-CATENIN 48 hours

92 - kDa

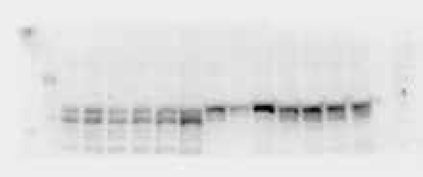

Total β-CATENIN 48 hours

45 - kDa

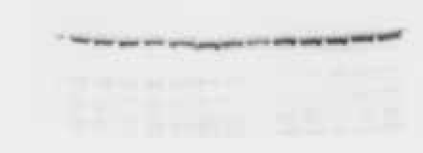

β-ACTIN 48 hours

92 - kDa

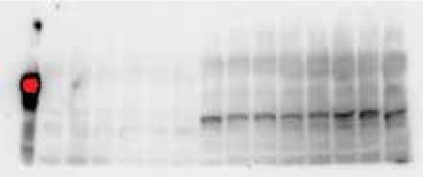

p-β-CATENIN 72 hours

92 - kDa

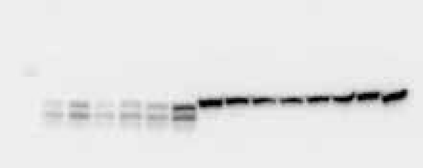

Active β-CATENIN 72 hours

92 - kDa

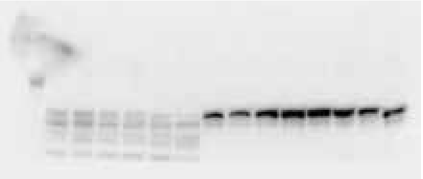

Total β-CATENIN 72 hours

45 - kDa

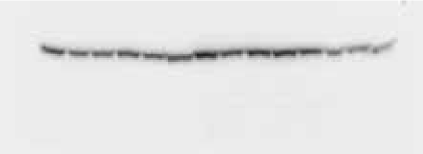

β-ACTIN 72 hours

92 - kDa

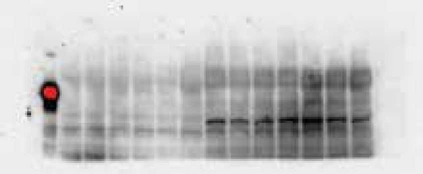

p-β-CATENIN 96 hours

92 - kDa

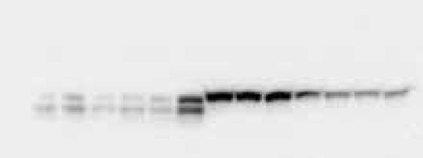

Active β-CATENIN 96 hours

92 - kDa

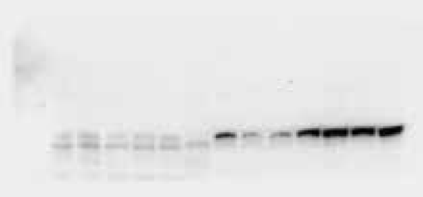

Total β-CATENIN 96 hours

45 - kDa

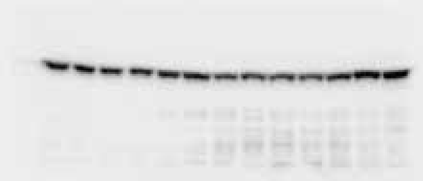

β-ACTIN 96 hours

K. Uncropped Western Blots for Supplementary Fig. S3B

38,
36 -
kDa

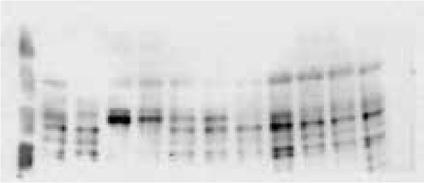

Cyclin D1 6 hours

36 -
kDA

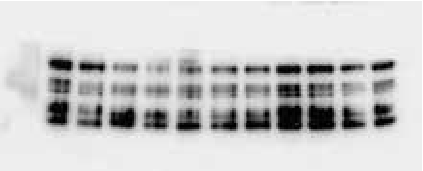

PCNA 6 hours

45 -
kDa

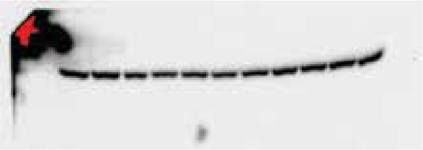

β-ACTIN 6 hours

38,
36 -
kDa

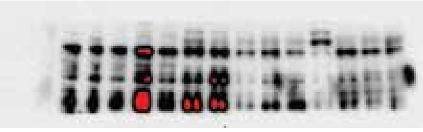

Cyclin D1 12 hours

36 -
kDA

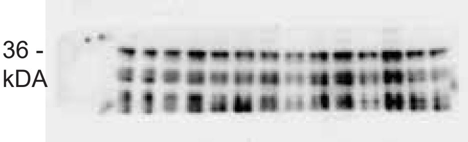

PCNA 12 hours

45 -
kDa

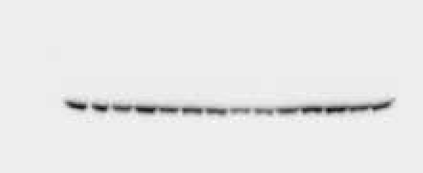

β-ACTIN 12 hours

38,
36 -
kDa

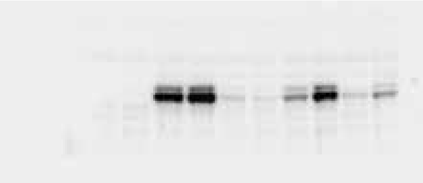

Cyclin D1 24 hours

36 -
kDA

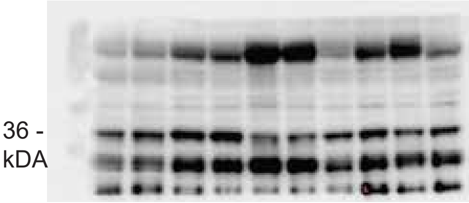

PCNA 24 hours

45 -
kDa

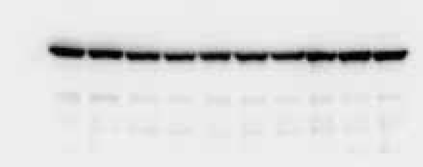

β-ACTIN 24 hours

38,
36 -
kDa

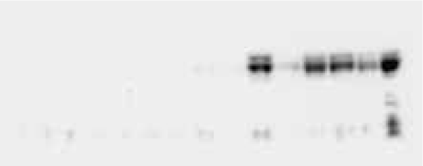

Cyclin D1 48 hours

36 -
kDA

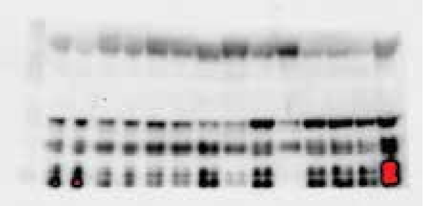

PCNA 48 hours

45 -
kDa

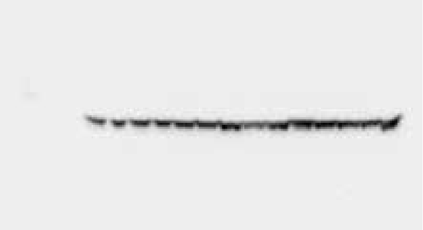

β-ACTIN 48 hours

38,
36 -
kDa

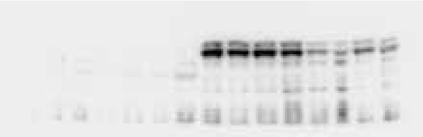

Cyclin D1 72 hours

36 -
kDA

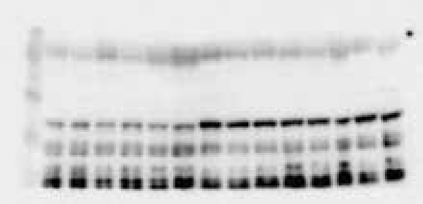

PCNA 72 hours

45 -
kDa

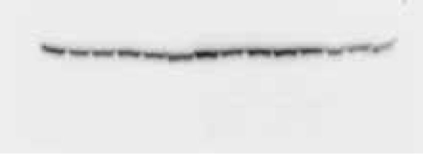

β-ACTIN 72 hours

38,
36 -
kDa

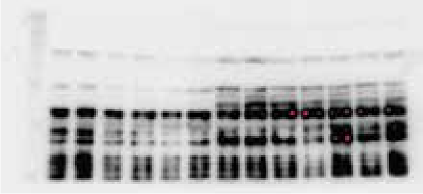

Cyclin D1 96 hours

36 -
kDA

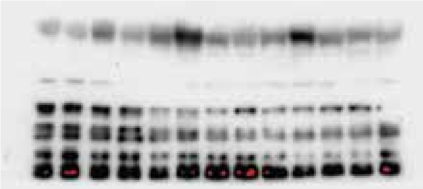

PCNA 96 hours

45 -
kDa

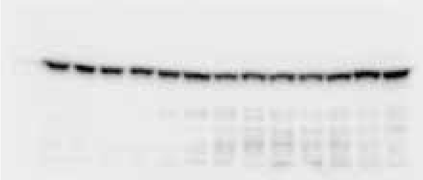

β-ACTIN 96 hours

L. Uncropped Western Blots for Supplementary Fig. S4D

92 -
kDa

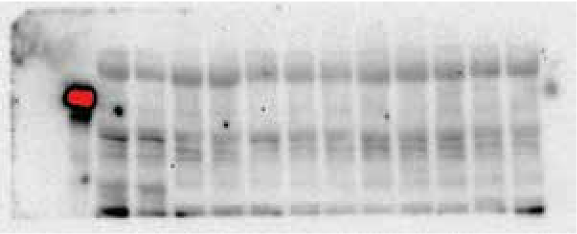

p-β-CATENIN 12 hours

92 -
kDa

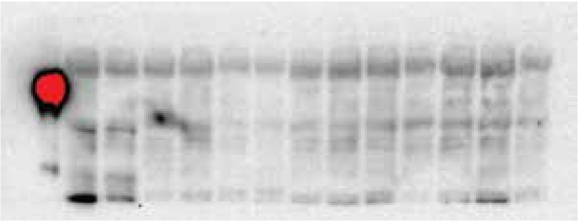

p-β-CATENIN 24 hours

92 -
kDa

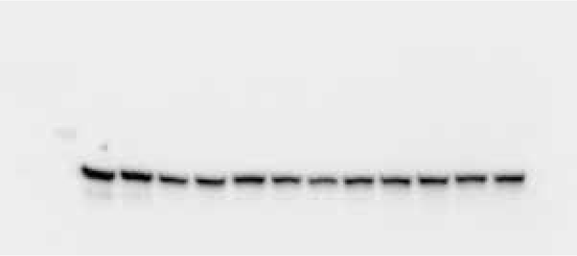

Active β-CATENIN 12 hours

92 -
kDa

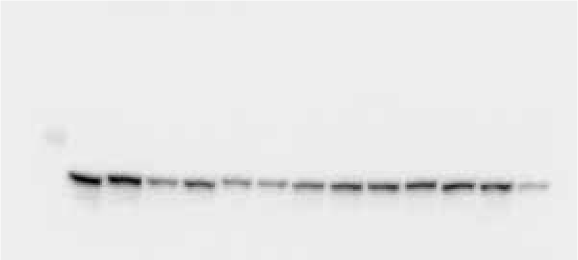

Active β-CATENIN 24 hours

92 -
kDa

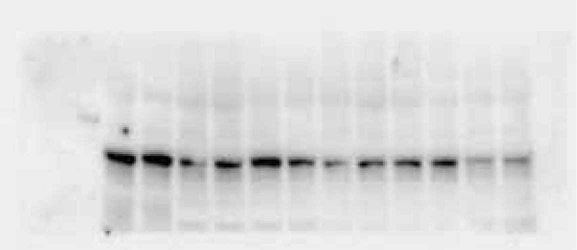

Total β-CATENIN 12 hours

92 -
kDa

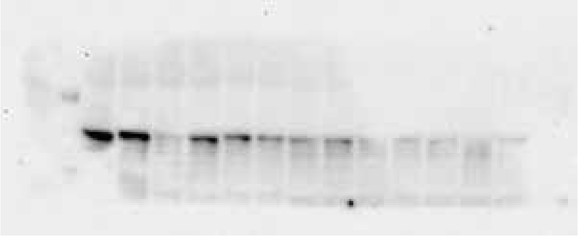

Total β-CATENIN 24 hours

45 -
kDa

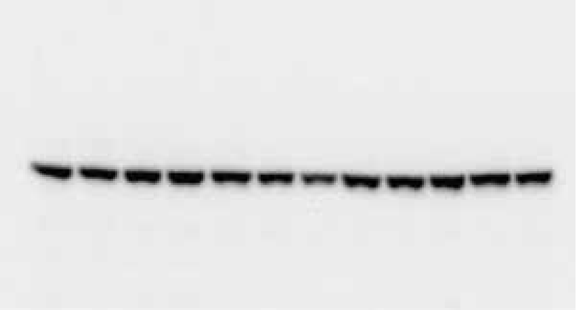

β-ACTIN 12 hours

45 -
kDa

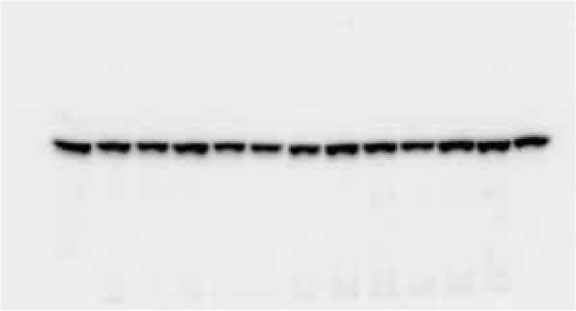

β-ACTIN 24 hours

92 -
kDa

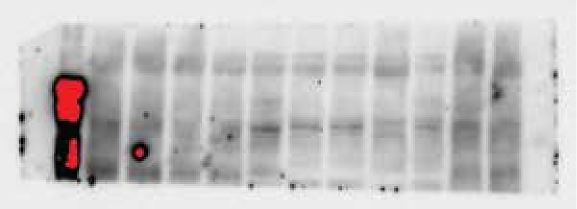

p-β-CATENIN 48 hours

92 -
kDa

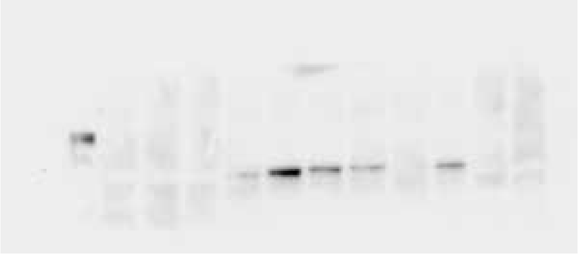

Total β-CATENIN 48 hours

92 -
kDa

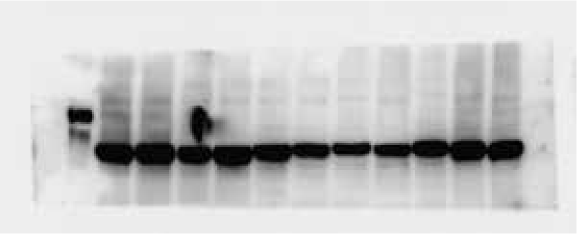

Active β-CATENIN 48 hours

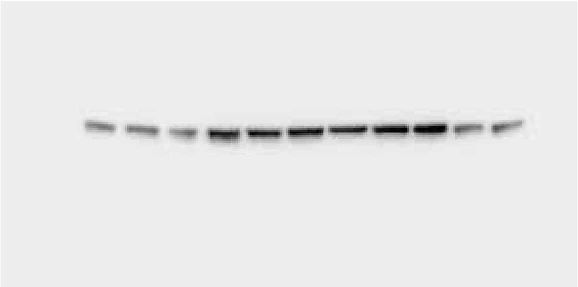

β-ACTIN 48 hours

M. Uncropped Western Blots for Supplementary Fig. S4E

38,
36 -
kDa

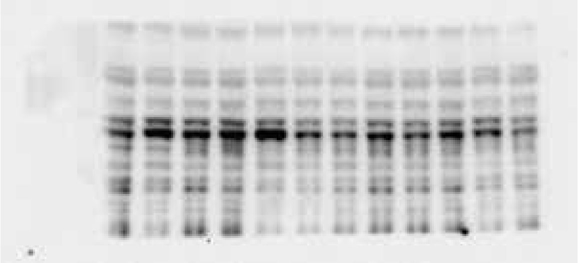

Cyclin D1 12 hours

38,
36 -
kDa

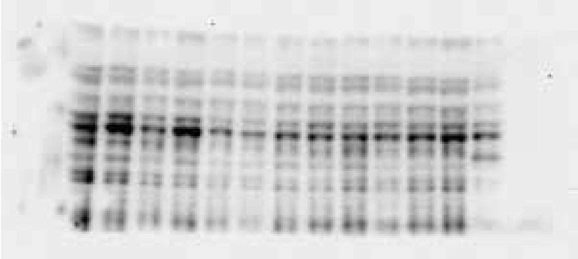

Cyclin D1 24 hours

36 -
kDA

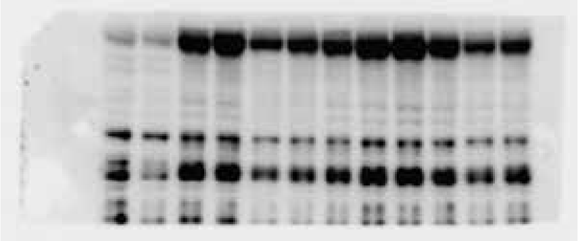

PCNA 12 hours

36 -
kDA

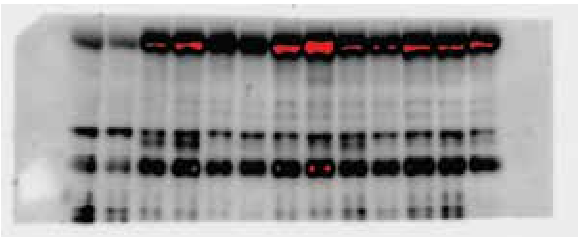

PCNA 24 hours

45 -
kDa

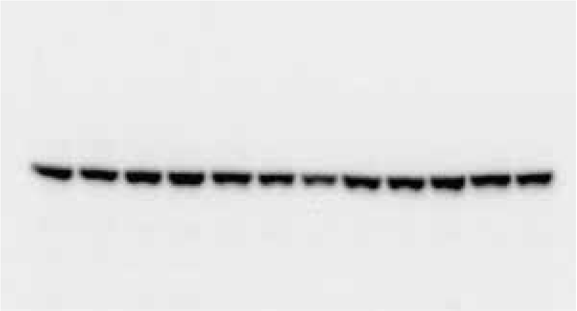

β-ACTIN 12 hours

45 -
kDa

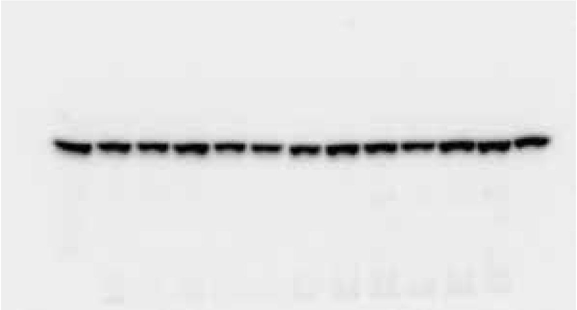

β-ACTIN 24 hours

38,
36 -
kDa

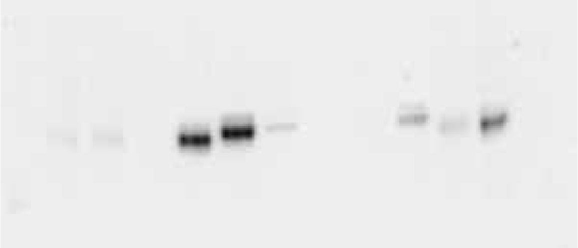

Cyclin D1 48 hours

36 -
kDA

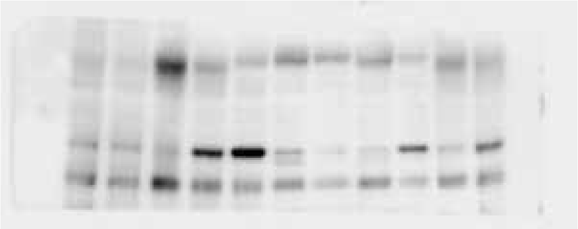

PCNA 48 hours

45 -
kDa

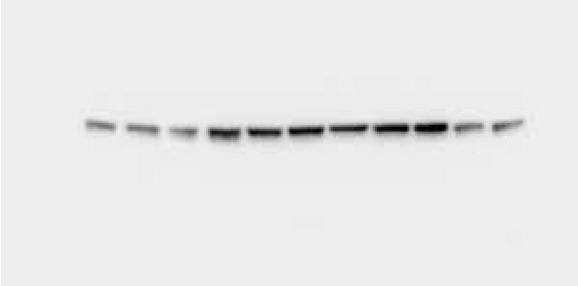

β-ACTIN 48 hours

N. Uncropped Western Blots for Supplementary Fig. S5

120 - kDa

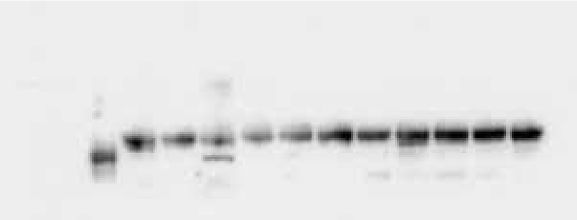

SIRT1

44 - kDa

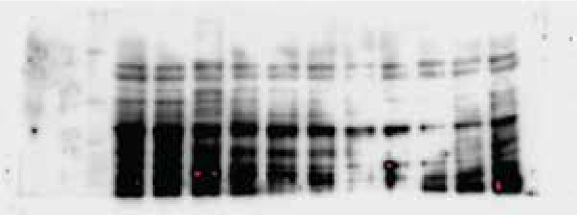

SIRT3

36 - kDa

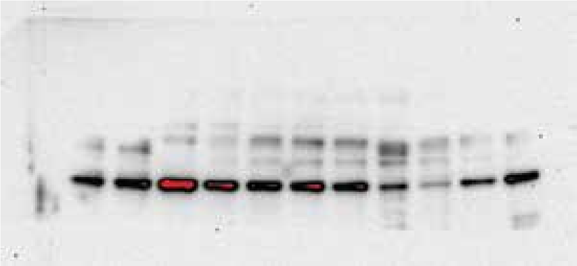

SIRT6

45 - kDa

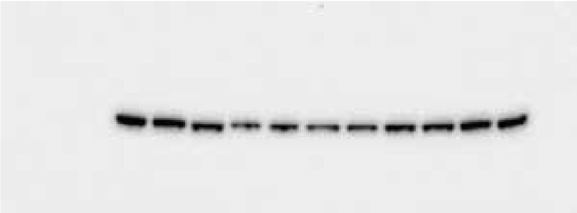

β-ACTIN

O. Uncropped Western Blots for Supplementary Fig. S6A

54 -
46 -
kDa

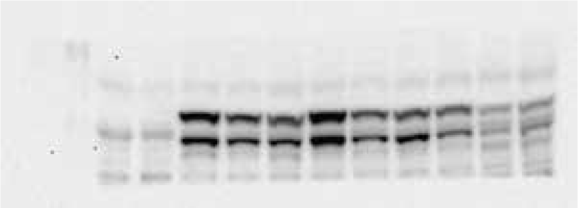

pJNK 0.5 hour

54 -
46 -
kDa

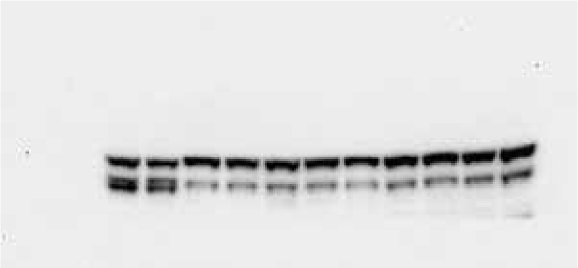

JNK 0.5 hour

45 -
kDa

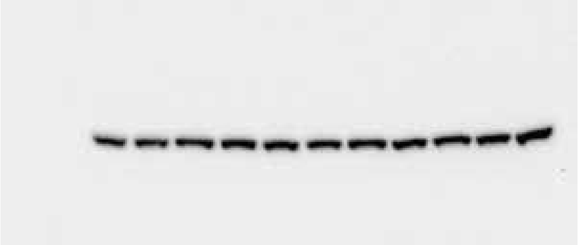

β-ACTIN 0.5 hour

54 -
46 -
kDa

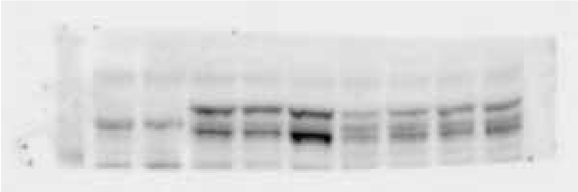

pJNK 6 hour

54 -
46 -
kDa

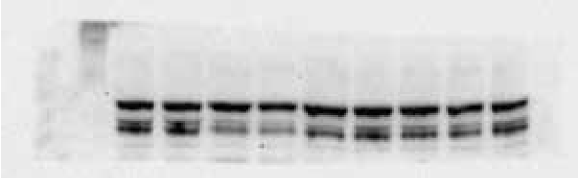

JNK 6 hour

45 -
kDa

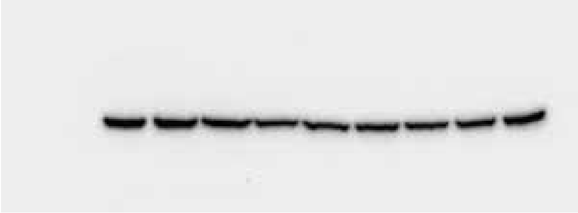

β-ACTIN 6 hour

54 -
46 -
kDa

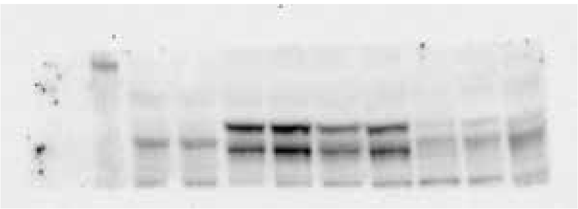

pJNK 12 hour

54 -
46 -
kDa

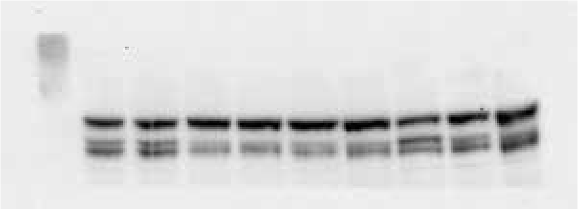

JNK 12 hour

45 -
kDa

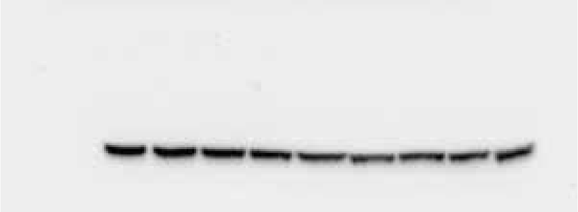

β-ACTIN 12 hour

54 -
46 -
kDa

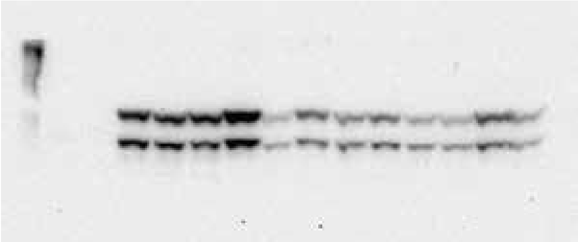

pJNK 24 hour

54 -
46 -
kDa

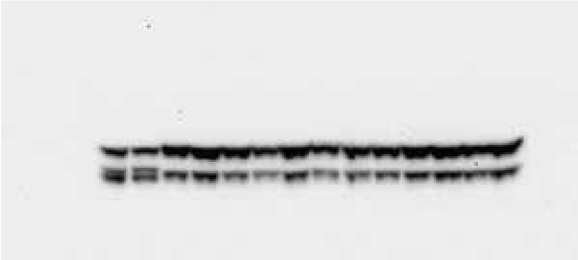

JNK 24 hour

45 -
kDa

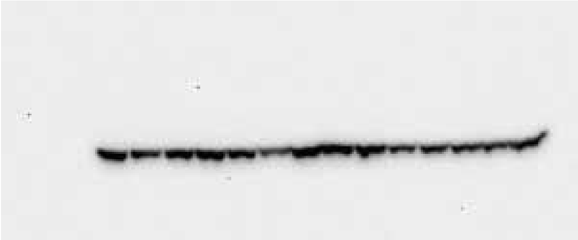

β-ACTIN 24 hour

P. Uncropped Western Blots for Supplementary Fig. S6B

54 -
46 -
kDa

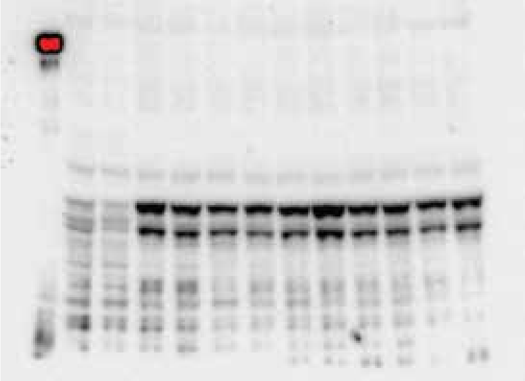

pJNK 12 hours

54 -
46 -
kDa

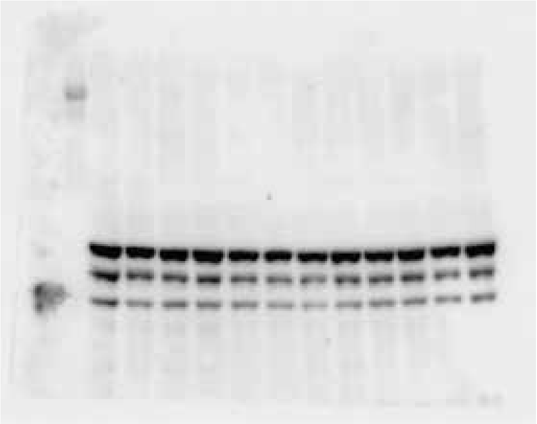

JNK 12 hours

45 -
kDa

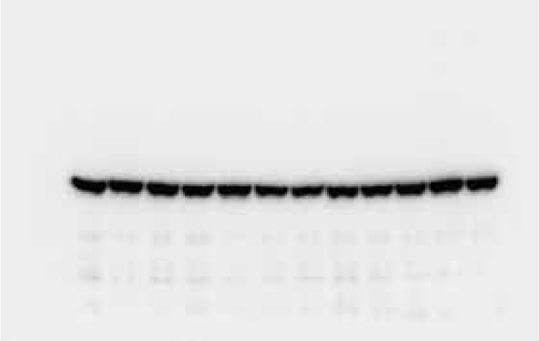

β-ACTIN 12 hours

54 -
46 -
kDa

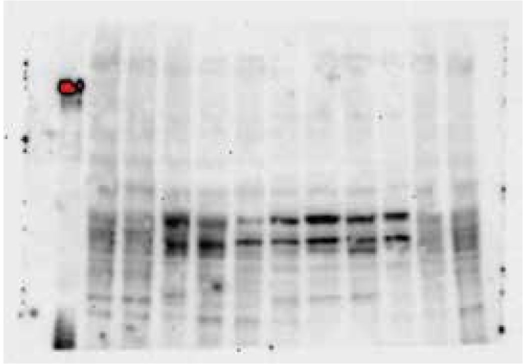

pJNK 48 hours

54 -
46 -
kDa

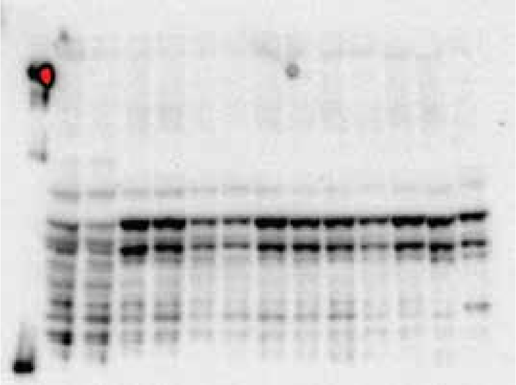

pJNK 24 hours

54 -
46 -
kDa

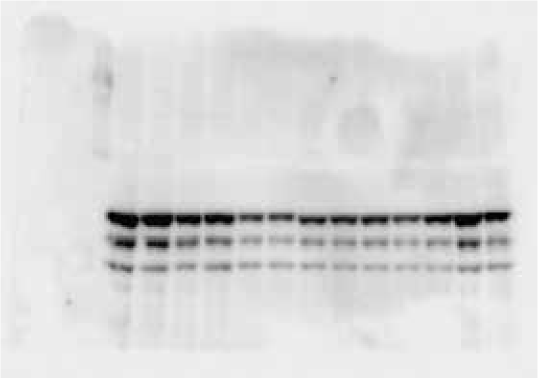

JNK 24 hours

45 -
kDa

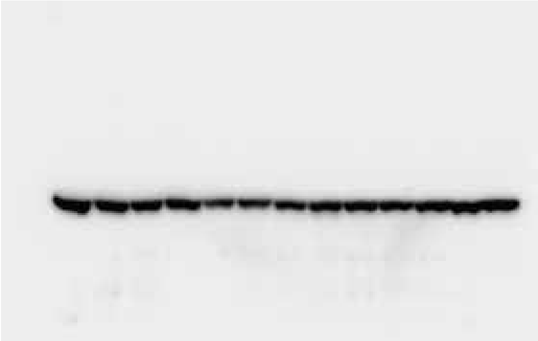

β-ACTIN 24 hours

54 -
46 -
kDa

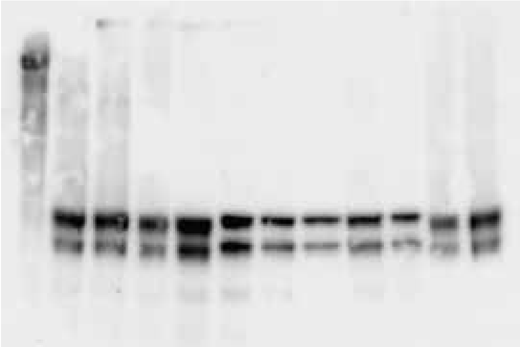

JNK 48 hours

45 -
kDa

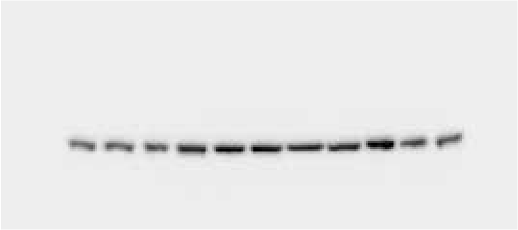

β-ACTIN 48 hours
